# Investigations of the Ligand Electronic Effects on α-Diimine Nickel(II) Catalyzed Ethylene Polymerization

**DOI:** 10.3390/polym8020037

**Published:** 2016-01-29

**Authors:** Lihua Guo, Shengyu Dai, Changle Chen

**Affiliations:** 1 School of Chemistry and Chemical Engineering, Qufu Normal University, Qufu 273165, China; guolihua-qfnu@139.com; 2 Key Laboratory of Soft Matter Chemistry, Chinese Academy of Sciences, Department of Polymer Science and Engineering, University of Science and Technology of China, Hefei 230026, China; daiyu@mail.ustc.edu.cn

**Keywords:** α-diimine, Nickel, dibenzhydryl, ethylene polymerization, electronic effect, cocatalyst

## Abstract

The synthesis and characterization of a series of dibenzhydryl-based α-diimine Ni(II) complexes bearing a range of electron-donating or -withdrawing groups are described. Polymerization with ethylene is investigated in detail, involving the activator effect, influence of polymerization conditions on catalyst activity, thermal stability, polymer molecular weight and melting point. All of these Ni(II) complexes show great activity (up to 6 × 10^6^ g of PE (mol of Ni)^−1^*·*h^−1^), exceptional thermal stability (stable at up to 100 °C) and generate polyethylene with very high molecular weight (*M*_n_ up to 1.6 × 10^6^) and very narrow molecular weight distribution. In the dibromo Ni(II) system, the electronic perturbations exhibit little variation on the ethylene polymerization. In the Ni(acac) system, dramatic ligand electronic effects are observed in terms of catalytic activity and polyethylene molecular weight.

## 1. Introduction

The initial reports by Brookhart and coworkers showed that complexes of Ni(II) and Pd(II) bearing sterically hindered α-diimine ligands could generate high-molecular-weight polymer with high catalytic activity in ethylene polymerization and incorporate polar comonomers into polyolefins for the Pd(II) catalyst [1,2]. A key insight from these early studies is that bulky *ortho*-aryl substituents in the α-diimine ligand could retard the associative chain transfer process by sterically blocking the access of monomers to the metal axial positions [1,3], which is essential to achieve high polymerization activity and high polymer molecular weight. Additionally, the high catalytic activities can be achieved by the destabilization of the ground state species from steric effect. The degree of branching and branching distribution, which is regulated by the competition between chain propagation and chain walking, can be changed through the tailoring of ligand structures. Since Brookhart’s seminal discovery, tremendous efforts have been made to explore new late-transition metal catalysts for olefin polymerization and copolymerization with polar monomers [3,4,5,6,7,8,9,10,11,12,13,14,15]. Despite these exciting features, one drawback of these catalysts is their low thermal stability, which greatly hinders their potential industrial applications (70–110 °C) [16]. It has been reported that α-diimine Pd(II) and Ni(II) catalysts undergo rapid decomposition over 60 °C because of increased associative chain transfer, C–H activation of the ligand, potential decomposition via *in situ*–generated metal hydride species and bis-ligation during the polymerization process [17,18,19,20].

**Scheme 1:**
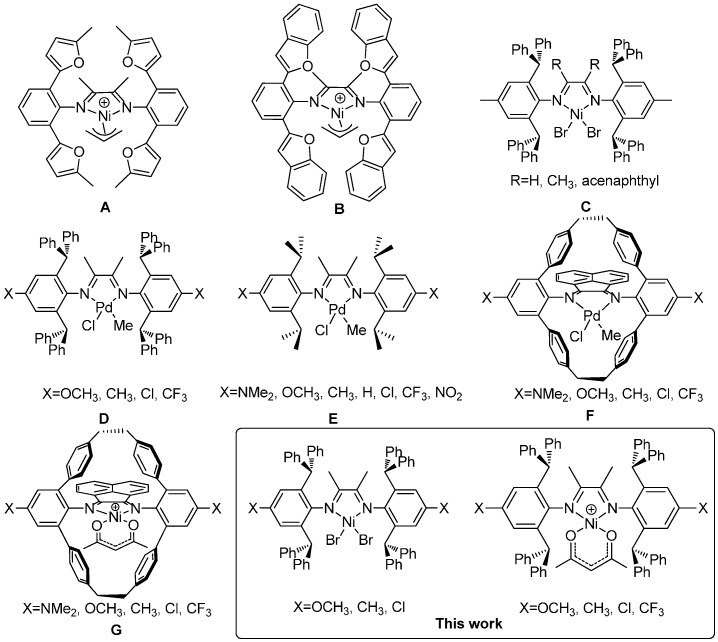
Modifications on the Pd(II) and Ni(II) complexes bearing α-diimine ligands.

Modifications of the *N*-aryl substituents and the ligand backbone have been very important to improve the thermal stability of α-diimine Pd(II) and Ni(II) catalysts. For example, Ionkin *et al.* reported that *o*-benzofuran-substituted α-diimine Ni(II) complexes (Scheme 1**A,B**) could maintain high activity even at 150 °C and generate high-molecular-weight polyethylene at 70 °C [21]. Subsequently, some cyclophane-based catalysts were shown to produce polyethylene with high activities and enhanced thermal stability [22,23,24,25,26]. In addition, catalysts bearing camphorquinone-derived ligands displayed moderate stability at up to 80 °C [20,27]. Recently, dibenzhyldryl-derived ligand frameworks were used to generate highly stable catalysts [28,29,30]. For example, Long *et al.* reported the studies of α-diimine Ni(II) catalysts (Scheme 1**C**) containing dibenzhydryl moiety, which maintained high activities at temperatures up to 100 °C [31,32]. Our group showed that α-diimine Pd(II) complexes (Scheme 1**D**) bearing the dibenzhydryl moiety displayed high thermal stability and high activity in ethylene (co)polymerization, producing semicrystalline polyethylene and ethylene/methyl acrylate copolymer with high molecular weight and low branching density [33]. We also investigated the ligand electronic effect in that system.

It was demonstrated that the ligand electronic effect played an important role on the polymerization properties in α-diimine catalyst systems. Guan *et al.* reported that higher polyethylene molecular weight and more linear topology were obtained with α-diimine Pd(II) catalysts (Scheme 1**E**) bearing electron-donating substituents. Catalysts bearing electron-donating substituents produced copolymers with higher methyl acrylate incorporation in ethylene-methyl acrylate copolymerization. Electron-donating substituents also led to more stable catalysts [34,35]. The same group studied the ligand electronic effect on a family of cyclophane-based α-diimine Pd(II) catalysts (Scheme 1**F**) [36]. The Pd(II) catalysts with electron-withdrawing substituents generated polymers with higher molecular weight than the electron-donating analogues, which was different from the previously studied acyclic α-diimine Pd(II) catalysts. Interestingly, there have been very few studies concerning the ligand electronic effects on α-diimine Ni(II) catalysts. Guan *et al.* studied some cyclophane-based α-diimine Ni(II) catalysts bearing different electron-donating or -withdrawing substituents, which showed very similar properties in ethylene polymerization (Scheme 1**G**) [36]. In this work, we synthesized and characterized a series of α-diimine Ni(II) complexes **1a**–**1c** and **2a**–**2d** bearing the dibenzhydryl moiety and a range of electron withdrawing and donating substituents. The ligand electronic effects upon the catalytic activity and thermal stability, along with analysis of polymer molecular weight and melting points, were investigated.

## 2. Experimental Section

### 2.1. General Information

All manipulations of air- and moisture-sensitive materials were performed under a dry Nitrogen atmosphere using standard Schlenk techniques or in a glove-box. Deuterated solvents for NMR were dried and distilled prior to use. Nitrogen was purified by passing through a MnO oxygen-removal column and an activated 4 Å molecular sieve column. Ethylene was purified by passing through an Agilent oxygen/moisture trap. The ^1^H and ^13^C NMR spectra were recorded on a Bruker Ascend™ 400 spectrometer (Bruker, Karlsruhe, Germany) at ambient temperature unless otherwise stated. The chemical shifts of the ^1^H and ^13^C NMR spectra (Bruker, Karlsruhe, Germany) were referenced to TMS. The ^19^F chemical shifts are reported relative to external CFCl_3_. Coupling constants are in Hz. Elemental analysis was performed by the Analytical Center of the University of Science and Technology of China. Mass spectra were recorded on a P-SIMS-Gly of Bruker Daltonics Inc. (EI+, Bruker Daltonics Inc., Billerica, USA). X-ray Diffraction data were collected at 298(2) K on a Bruker Smart CCD area detector (Bruker, Karlsruhe, Germany) with graphite-monochromated Mo Kα radiation (λ = 0.71073 Å). Gel permeation chromatography (GPC) was carried out at 150 °C by a PL-GPC 220 high-temperature gel permeation chromatography (PL, Shropshire, UK). The 1,2,4-Trichlorobenzene (TCB) was used as solvent at a flow rate of 1.0 mL·min^−1^, and the calibration was made using polystyrene standard and was corrected for linear polyethylene by universal calibration using the Mark–Houwink parameters of Rudin: *K* = 1.75 × 10*^−^*^2^ cm^3^/g and *R* = 0.67 for polystyrene and *K* = 5.90 × 10*^−^*^2^ cm^3^/g and *R* = 0.69 for polyethylene. Dichloromethane, toluene, THF, and hexanes were purified by solvent purification systems. The α-diimine ligands were prepared according to reported procedures [33]. All other reagents were purchased from commercial sources and used without purification.

### 2.2. Standard Procedure for the Synthesis of Complexes **1a–1c**

All complexes were prepared in a similar manner by the reaction of (DME)NiBr_2_ with the corresponding ligand in dichloromethane. A typical synthetic procedure for complexes **1a**–**1c** is as follows: ligand (1.0 mmol) and (DME)NiBr_2_ (0.31 g, 1.0 mmol) (DME = 1,2-dimethoxyethane) were stirred in 10 mL of CH_2_Cl_2_ overnight at room temperature. The solvent was removed, and the resulting powder was washed with hexane (10 mL × 2) and dried under vacuum to obtain a brown solid.

**(°^Me^N^N)NiBr_2_** **(1a)** (1.1 g, 93%): MALDI-TOF-MS (m/z): calcd. for C_70_H_60_BrN_2_NiO_2_: 1099.3171, found: 1099.5005 [M–Br]^+^. Anal. Calcd. for C_70_H_60_Br_2_N_2_NiO_2_: C, 71.27; H, 5.13; N, 2.37; Found: C, 70.98; H, 5.34; N, 2.28.

**(^Me^N^N)NiBr_2_** **(1b)** (1.0 g, **87%**): MALDI-TOF-MS (m/z): calcd. for C_70_H_60_BrN_2_Ni: 1067.3273, found: 1067.5190 [M–Br]^+^. Anal. Calcd. for C_70_H_60_Br_2_N_2_Ni: C, 73.25; H, 5.27; N, 2.44; Found: C, 73.11; H, 5.31; N, 2.49.

**(^Cl^N^N)NiBr_2_** **(1c)** (1.0 g, 89%): HRMS (m/z): calcd. for C_68_H_54_Cl_2_N_2_Ni: 1028.2988, found: 1028.4939 [M–2Br]^2+^. Anal. Calcd. for C_68_H_54_Br_2_Cl_2_N_2_Ni: C, 68.72; H, 4.58; N, 2.36; Found: C, 68.43; H, 4.79; N, 2.29.

### 2.3. Standard Procedure for the Synthesis of Complexes **2a**–**2d**

A typical synthetic procedure for complexes **2a**–**2d** is as follows: ligand (1.0 mmol), trityltetrkis(pentafluorophenyl)borate ([Ph_3_C][B(C_6_F_5_)_4_]) (1.0 mmol), and Ni(acac)_2_ (1.0 mmol) were stirred in 10 mL of dry CH_2_Cl_2_ overnight at room temperature. The mixture was concentrated in vacuum to 3 mL, and added with diethyl ether (10 mL) and hexane (15 mL). The mixture was filtered, and the solid was washed with diethyl ether (10 mL) and pentane (15 mL). Subsequent drying under vacuum afforded **2a**–**2d** as dark-red solids at 83%–88% yields.

**[(°^Me^N^N)Ni(acac)][B(C_6_F_5_)_4_]** **(2a)** dark red solid. Yield 88% (1.58 g). ^1^H NMR (CDCl_3_, 400 MHz): δ 7.25–7.18 (m, 32H, CH*Ph*_2_), 7.14–7.11 (m, 8H, CH*Ph*_2_), 6.61 (s, 4H, N–*aryl*), 5.99 (s, 4H, C*H*Ph_2_), 5.11 (s, 1H, acac–C*H*), 3.62 (s, 6H, aryl–OC*H*_3_), 1.46 (s, 6H, acac–C*H*_3_), 0.71 (s, 6H, N=C*Me*). ^13^C NMR (100 MHz, CDCl_3_): δ 186.80 (N=*C*Me), 179.92 (N=*C*Me), 159.35 (O–*C*^p−Ar^), 140.92 (*Ar*), 140.78 (*Ar*), 129.33 (*Ar*), 129.31 (*Ar*), 129.16 (*Ar*), 128.72 (*Ar*), 127.50 (*Ar*), 114.94 (*Ar*), 102.47 (*Ar*), 55.40 (O*C*H_3_), 52.94 (*C*H), 24.60 (acac–*C*H_3_), 19.33 (N=C–*Me*). Anal. Calcd. for C_99_H_67_BF_20_N_2_NiO_4_: C, 66.13; H, 3.76; N, 1.56; Found: C, 66.17; H, 3.82; N, 1.61.

**[(^Me^N^N)Ni(acac)][B(C_6_F_5_)_4_]** **(2b)** dark red solid. Yield 83% (1.47 g). ^1^H NMR (CDCl_3_, 400 MHz): δ 7.25–7.17 (m, 32H, CH*Ph*_2_), 7.12–7.09 (m, 8H, CH*Ph*_2_), 6.89 (s, 4H, N–*aryl*), 6.00 (s, 4H, C*H*Ph_2_), 5.07 (s, 1H, acac–C*H*), 2.23 (s, 6H, aryl–C*H*_3_), 1.41 (s, 6H, acac–C*H*_3_), 0.72 (s, 6H, N=C*Me*). ^13^C NMR (100 MHz, CDCl_3_): δ 186.66 (N=*C*Me), 179.43 (N=*C*Me), 141.13 (*Ar*), 141.02 (*Ar*), 138.98 (*Ar*), 136.67 (*Ar*), 136.20 (*Ar*), 130.00 (*Ar*), 129.35 (*Ar*), 129.29 (*Ar*), 129.20 (*Ar*), 128.69 (*Ar*), 127.71 (*Ar*), 127.39 (*Ar*), 102.37 (*Ar*), 52.77 (*C*H), 24.54 (acac–*C*H_3_), 21.73 (aryl–*C*H_3_), 19.33 (N=C–*Me*). Anal. Calcd. for C_99_H_67_BF_20_N_2_NiO_2_: C, 67.33; H, 3.82; N, 1.59; Found: C, 67.26; H, 3.81; N, 1.60.

**[(^Cl^N^N)Ni(acac)][B(C_6_F_5_)_4_]** **(2c)** dark red solid. Yield 87% (1.57 g). ^1^H NMR (CDCl_3_, 400 MHz): δ 7.26–7.21 (m, 32H, CH*Ph*_2_), 7.08–7.09 (m, 12H, CH*Ph*_2_ and N–*aryl*), 5.98 (s, 4H, C*H*Ph_2_), 5.12 (s, 1H, acac–C*H*), 1.48 (s, 6H, acac–C*H*_3_), 0.74 (s, 6H, N=C*Me*). ^13^C NMR (100 MHz, CDCl_3_): δ 186.84 (N=*C*Me), 180.00 (N=*C*Me), 140.15 (*Ar*), 140.06 (*Ar*), 138.46 (*Ar*), 137.29 (*Ar*), 135.17 (*Ar*), 129.60 (*Ar*), 129.55 (*Ar*), 129.21 (*Ar*), 129.04 (*Ar*), 128.98 (*Ar*), 128.19 (*Ar*), 127.86 (*Ar*), 102.57 (*Ar*), 52.79 (*C*H), 24.71 (acac–*C*H_3_), 19.50 (N=C–*Me*). Anal. Calcd. for C_97_H_61_BCl_2_F_20_N_2_NiO_2_: C, 64.48; H, 3.40; N, 1.55; Found: C, 64.44; H, 3.36; N, 1.57.

**[(^CF^_3_N^N)Ni(acac)][B(C_6_F_5_)_4_]** **(2d)** dark red solid. Yield 88% (0.33 g). ^1^H NMR (CDCl_3_, 400 MHz): δ 7.38–7.23 (m, 36H, CH*Ph*_2_), 7.12–7.07 (m, 8H, CH*Ph*_2_ and N–*aryl*), 6.09 (s, 4H, C*H*Ph_2_), 5.12 (s, 1H, acac–C*H*), 1.41 (s, 6H, acac–C*H*_3_), 0.77 (s, 6H, N=C*Me*). ^13^C NMR (100 MHz, CDCl_3_): δ 186.94 (N=*C*Me), 179.94 (N=*C*Me), 141.72 (*Ar*), 139.94 (*Ar*), 139.92 (*Ar*), 138.09 (*Ar*), 129.73 (*Ar*), 129.16 (*Ar*), 129.12 (*Ar*), 129.02 (*Ar*), 128.37 (*Ar*), 128.07 (*Ar*), 126.23 (*Ar*), 126.20 (*Ar*), 102.65 (*Ar*), 52.93 (*C*H), 24.47 (acac–*C*H_3_), 19.59 (N=C–*Me*). ^19^F NMR (282 MHz, CDCl_3_): δ −62.81 (s, C*F*_3_), -132.53 (d, *J* = 14.1 Hz), −163.14 (t, *J* = 16.9 Hz), 166.78 (t, *J* = 15.6Hz). Anal. Calcd. for C_99_H_61_BF_26_N_2_NiO_2_: C, 63.45; H, 3.28; N, 1.49; Found: C, 63.53; H, 3.21; N, 1.42.

### 2.4. General Procedure for Ethylene Polymerization

In a typical experiment, a 350 mL glass thick-walled pressure vessel was charged with required amount of aluminum activator (MAO (methylaluminoxane), AlEt_2_Cl, Al(i-Bu)_3_), 48 mL toluene and a magnetic stir bar in the glovebox. The pressure vessel was connected to a high pressure polymerization line and the solution was degassed. The vessel was warmed to the desired temperature using an oil bath and allowed to equilibrate for 5 min. Then 1.0 μmol of nickel complex in 2 mL CH_2_Cl_2_ was injected into the vessel via syringe. With rapid stirring, the reactor was pressurized and maintained at 9.0 atm of ethylene. After a desired amount of polymerization time, the vessel was vented and the polymer was precipitated in acidified methanol (methanol/HCl = 50/1) and dried under vacuum at 50 °C for 24 h.

## 3. Results and Discussion

### 3.1. Synthesis and Characterization of the Ni(II) Complexes

**Scheme 2:**
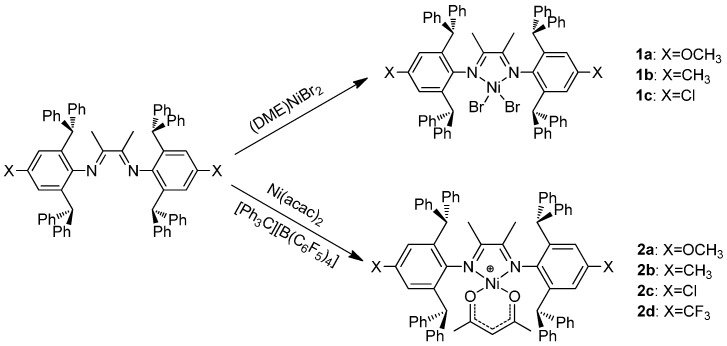
Synthesis of nickel complexes **1a**–**1c** and **2a**–**2d**.

The ligands were prepared using the literature procedure in high yields without using column chromatography [33]. Nickel dibromo complexes of α-diimine ligands are typical catalytic precursors in olefin polymerization [1]. It should be noted that complex **1b** with CH_3_ substituent has been studied by Long *et al.* (See [31]). This complex was prepared and studied in this work in order to obtain accurate comparisons in ethylene polymerization. Nickel dibromo complexes (**1a**–**1c**) were prepared by reacting the ligands with 1 equiv. of (DME)NiBr_2_ (Scheme 2). However, no conversion was achieved in the case of the electron-poor trifluoromethyl-substituted ligands. These Ni complexes were characterized by elemental analysis and mass spectrometry.

Alternatively, the cationic acetylacetonato (acac) complexes [Ni(α-diimine)(acac)][B(C_6_F_5_)_4_] (**2a**–**2d**) were successfully synthesized at 83%–88% yields using the literature procedure [36,37,38] from the reaction of the ligands with [Ph_3_C][B(C_6_F_5_)_4_] and Ni(acac)_2_ (Scheme 2). Complexes **2a**–**2d** are diamagnetic because of their square planar geometry at the Ni center, and were thus characterized by ^1^H and ^13^C NMR. These Ni complexes were also characterized by elemental analysis.

**Figure 1:**
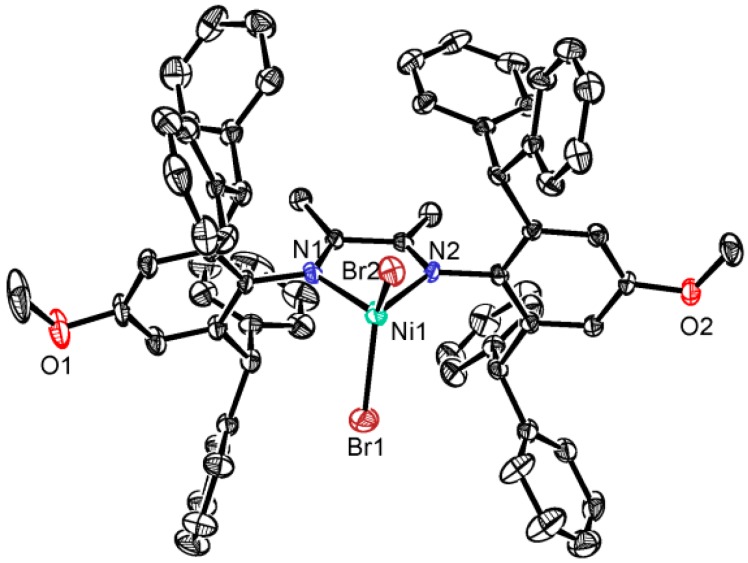
Molecular structure of complex **1a** (thermal ellipsoids are shown at the 30% probability level). Hydrogen atoms have been omitted for clarity. Solvent molecule (CH_2_Cl_2_) was also omitted.

A single crystal of complex **1a** was obtained by layering hexane onto the CH_2_Cl_2_ solution at room temperature. The molecular structure of **1a** was determined by X-ray diffraction analysis (Figure 1). In solid state, the nickel center adopts distorted tetrahedron geometry with a N1–Ni1–N2 angle of 81.1(2)° and a Br1–Ni1–Br2 angle of 123.45(5)°. The observed bond lengths are typical for Ni(II) α-diimine complexes with Ni1–Br1 = 2.3058(12) Å, Ni1–Br2 = 2.3163(11) Å, Ni1–N1 = 1.988(5) Å and Ni1–N2 = 1.986(5) Å. The Ni–Br and Ni–N bond distances in **1a** are slightly shorter than those in **1b** (Ni–Br: 2.3286 and 2.3351 Å; Ni–N: 1.9978 and 2.0020 Å) [31].

### 3.2. Ethylene Polymerization Studies

**Figure 2:**
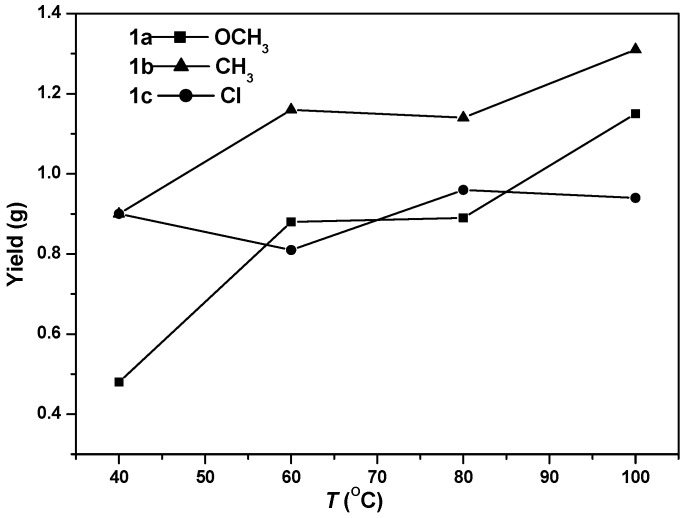
Polyethylene yield *versus* temperature for complexes **1a**–**1c** at 40, 60, 80 and 100 °C (Table 1).

All of the dibromo Ni(II) complexes (**1a**–**1c**) were highly active in ethylene polymerization when activated with organoaluminum activators (Table 1). For all of these complexes, the catalytic activities were increased with polymerization temperatures and the highest activity was observed at 100 °C (Table 1, Figure 2). Their activities are on the level of 10^6^ g of PE (polyethylene) (mol of Ni)^−1^·h^−1^ at all polymerization temperatures. These complexes represent one of the most active and thermally stable α-diimine Ni(II) catalysts in ethylene polymerization. At 100 °C for complexes **1a**–**1c**, the MAO activator gave higher polymerization activity, generating polyethylene with higher molecular weight and narrower molecular weight distribution than the AlEt_2_Cl activator (Table 1, Entries 4, 5, 9, 10, 14 and 15). Therefore, MAO was chosen as the activator in the dibromo Ni(II) system.

**Table 1:** Ethylene polymerization results with complexes **1a**–**1c**. ^a^

Entry	Cat.	Activator	*T* (°C)	Yield (g)	Act. ^b^	*M*_n_^c^ (*×*10^−4^)	PDI ^c^	Br ^d^	*T*_m_^e^ (°C)
1	**1a**	MAO	40	0.48	0.96	111	1.19	48	62.1
2	**1a**	MAO	60	0.88	1.76	126	1.22	53	53.7
3	**1a**	MAO	80	0.89	1.78	146	1.21	56	49.2
4	**1a**	MAO	100	1.15	2.30	125	1.44	62	41.4
5	**1a**	AlEt_2_Cl	100	0.79	1.58	103	1.88	59	47.6
6	**1b**	MAO	40	0.90	1.80	161	1.12	55	57.2
7	**1b**	MAO	60	1.16	2.32	159	1.25	59	46.0
8	**1b**	MAO	80	1.14	2.28	164	1.23	62	43.8
9	**1b**	MAO	100	1.31	2.62	154	1.40	66	39.1
10	**1b**	AlEt_2_Cl	100	1.24	2.48	152	1.55	62	42.8
11	**1c**	MAO	40	0.90	1.80	143	1.20	59	48.3
12	**1c**	MAO	60	0.81	1.62	147	1.25	65	39.8
13	**1c**	MAO	80	0.96	1.92	129	1.34	71	35.1
14	**1c**	MAO	100	0.94	1.88	121	1.57	74	34.9
15	**1c**	AlEt_2_Cl	100	0.39	0.78	80.4	1.79	65	37.9

^a^ Polymerization conditions: 1.0 μmol of Ni(II) complex; Al/Ni = 600; 48 mL toluene and 2 mL CH_2_Cl_2_; ethylene = 9 atm; time = 30 min. ^b^ Activity, 10^6^ g of PE (mol of Ni)^−1^·h^−1^; ^c^ PDI = polydispersity index, determined by GPC (gel permeation chromatography); ^d^ Br = branches per 1000 carbon, determined by ^1^H NMR; ^e^ Melting temperature, determined by DSC (differential scanning calorimetry).

Under almost all of the polymerization conditions, the molecular weight (*M*_n_) of the generated polyethylene is higher than one million, representing a big advantage of this catalyst system. Moreover, the molecular weight was only slightly decreased at elevated temperatures. For example, the *M*_n_ was slightly dropped from 164 × 10^4^ at 80 °C to 154 × 10^4^ at 100 °C for complex **1b**, and the melting point was decreased from 43.8 to 39.1°C (Table 1, Entries 8 and 9). These results suggest that the chain transfer is greatly suppressed by the bulky ligands even at elevated temperatures. However, the chain walking rate is increased with increasing temperature, leading to an obvious drop in polymer melting point. The resultant polyethylene obtained at 40 °C displayed a very narrow molecular weight distribution (PDI < 1.20) while relatively broad molecular weight distributions (PDI > 1.40) were observed at elevated temperatures. No clear correlation between the ligand electronic effects and the catalytic properties was observed in this system. This is similar with the cyclophane-based α-diimine Ni(II) system (Scheme 1**G**) [36].

The branching densities of the obtained polyethylenes were increased with polymerization temperatures, following the same trend as other α-diimine Ni(II) catalysts [3]. Interestingly, the branching densities were observed to be lower for the catalyst with electron-donating substituents. For example, with MAO at 100 °C, the branching density for polyethylene generated using catalyst **1a** is 62/1000C (Table 1, Entry 4), whereas branching density of 74/1000C is observed for catalyst **1c** (Table 1, Entry 14). In addition, activator AlEt_2_Cl led to slightly lower branching density than activator MAO.

Since the dibromo nickel catalyst with CF_3_ substituent cannot be prepared, the Ni(acac) system could provide more systematic comparisons (Table 2). Complex **2b** was selected to probe the influence of aluminum cocatalysts and polymerization conditions. In sharp contrast to complex **1b**, MAO was not able to activate complex **2b** for ethylene polymerization at 40 or 80 °C (Table 2, entries 1 and 3). Triisobutylaluminum (TIBA) and AlEtCl_2_ cocatalysts were not effective either (Table 2, entries 2, 4 and 5). Luckily, cocatalyst AlEt_2_Cl was able to activate **2b** for ethylene polymerization. Similar to complex **1b**, the activity of complex **2b** was increased with temperature in the range of 40–100 °C (Table 2, entries 6–9). However, the increase in activity with temperature in complex **2b** is much more dramatic than complex **1b**. The activities and the polyethylene molecular weight of **2b** are similar to the cases of **1b**. These results suggest that it is difficult to activate the bulky catalysts in the Ni(acac) system. The right cocatalyst and high polymerization temperatures are necessary to generate active catalytic species.

**Table 2:** Ethylene polymerization results with complexes **2a**–**2d**. ^a^

Entry	Cat.	Activitor	*T* (°C)	Yield (g)	Act. ^b^	*M*_n_^c^ (*×*10^−4^)	PDI ^c^	Br ^d^	*T*_m_^e^ (°C)
1	**2b**	MAO	40	trace	−	−	−		−
2	**2b**	Al(i-Bu)_3_	40	trace	−	−	−		−
3	**2b**	MAO	80	trace	−	−	−		−
4	**2b**	Al(i-Bu)_3_	80	trace	−	−	−		−
5	**2b**	AlEtCl_2_	100	trace	−	−	−		−
6	**2b**	AlEt_2_Cl	40	0.53	1.06	115	1.26	47	70.5
7	**2b**	AlEt_2_Cl	60	0.86	1.72	130	1.44	51	62.6
8	**2b**	AlEt_2_Cl	80	1.43	2.86	145	1.35	63	53.5
9	**2b**	AlEt_2_Cl	100	1.88	3.76	157	1.52	66	45.3
10	**2a**	AlEt_2_Cl	100	1.07	2.14	60.6	1.75	61	46.0
11	**2c**	AlEt_2_Cl	100	1.40	2.80	81.0	1.79	63	39.9
12	**2d**	AlEt_2_Cl	100	3.09	6.18	57.5	2.14	62	41.5

^a^ Polymerization conditions: 1.0 μmol of Ni(II) complex; Al/Ni = 600; 48 mL toluene and 2 mL CH_2_Cl_2_; ethylene = 9 atm; time = 30 min; ^b^ Activity, 10^6^ g of PE (mol of Ni)^−1^ h^−1^; ^c^ PDI = polydispersity index, determined by GPC (gel permeation chromatography); ^d^ Br = branches per 1000 carbon, determined by ^1^H NMR; ^e^ Melting temperature determined by DSC (differential scanning calorimetry).

Quite surprisingly, complex **2d** containing the electron-withdrawing CF_3_ group showed the highest activity of 6.18 × 10^6^ g of PE (mol of Ni)^−1^·h^−1^ (Table 2, Entry 12), which is almost three times as much as that of **2a** (2.14 × 10^6^ g of PE (mol of Ni)^−1^·h^−1^, Table 2, Entry 10) and twice as much as that of **2b** (3.76 × 10^6^ g of PE (mol of Ni)^−1^·h^−1^, Table 2, Entry 9) and **2c** (2.80 × 10^6^ g of PE (mol of Ni)^−1^·h^−1^, Table 2, Entry 11). However, complex **2d** generated polyethylene with the lowest molecular weight (*M*_n_ = 57.5 × 10^4^) among all complexes. Complex **2b** produced polyethylene with the highest molecular weight (157 × 10^4^), which is *ca*. twice as much as that by **2c** (81.0 × 10^4^) and three times as much as that by **2a** (60.6 × 10^4^) and **2d**. Complex **2a** bearing the electron-donating OMe group produced polyethylene with the highest melting point. However, the melting point is only slightly influenced by the ligand electronic effect. The molecular weight distribution of the polyethylene produced by the Ni(acac) system with the AlEt_2_Cl activator is broader than that of the dibromo Ni(II)/MAO system. The polymerization at 100 °C revealed the following trends in activity: CF_3_ > CH_3_ > Cl > OCH_3_, molecular weight: CH_3_ > Cl > OCH_3_ > CF_3_ and melting points: OCH_3_ > CH_3_ > CF_3_ > Cl. Despite the much more dramatic differences in ethylene polymerization in the Ni(acac) system compared with the dibromo nickel system, no clear trend was observed. Similar with that observed in complexes **1a**–**1c**, the polyethylene branching density was increased with polymerization temperature for complex **2b**. There was no obvious trend for branching density among complexes **2a**–**2d**. It is interesting to note the different performances between the dibromo nickel system and the Ni(acac) system. Theoretically, both systems should perform similarly after MAO or Et_2_AlCl activation, because of the same ligand structures and, correspondingly, the same active species. The differences we observed might originate from the differences in the activation/initiation processes or the different counter-anions in the two systems. Future work is required to better understand the ligand electronic effect in the α-diimine nickel catalyst system.

## 4. Conclusions

In conclusion, a series of dibenzhydryl-based α-diimine nickel complexes bearing a range of electron-donating and -withdrawing substituents were prepared. The aim is to systematically investigate the ligand electronic effects on the ethylene polymerization in this class of catalysts. The dibromo nickel-catalyzed ethylene polymerization was relatively insensitive to the electronic perturbation. However, electronic effects in the Ni(acac) system were pronounced, although no discernible trend was observed. The CF_3_-substituted complex **2d** exhibited exceptionally high activity and stability at elevated temperatures. All of these complexes were highly active in ethylene polymerization with activities on the level of 10^6^ g of PE (mol of Ni)^−1^·h^−1^, generating polyethylene with molecular weight larger than one million. These complexes also showed great thermal stability, maintaining high activity and high polyethylene molecular weight at up to 100 °C.

## Supplementary Materials

Supplementary materials can be found at www.mdpi.com/2073-4360/8/2/37/s1. NMR spectra of the complexes **2a**–**2d** (Figures S1–S9), MS of the complexes **1a**-**1c** (Figures S10–S12), crystal data of complex **1a** (Tables S1–S3) and the ^1^H NMR, GPC and DSC curves of polyethylene samples (Figures S13–S24).

## References

[1] Johnson L.K., Killian C.M., Brookhart M. (1995). New Pd (II)- and Ni (II)-based catalysts for polymerization of ethylene and α-olefins. J. Am. Chem. Soc..

[2] Killian C.M., Tempel D.J., Johnson L.K., Brookhart M. (1996). Living polymerization of α-olefins using Ni^II^-α-diimine catalysts. Synthesis of new block polymers based on α-olefins. J. Am. Chem. Soc..

[3] Ittel S.D., Johnson L.K., Brookhart M. (2000). Late-metal catalysts for ethylene homo- and copolymerization. Chem. Rev..

[4] Nakamura A., Ito S., Nozaki K. (2009). Coordination—Insertion copolymerization of fundamental polar monomers. Chem. Rev..

[5] Camacho D.H., Guan Z. (2010). Designing late-transition metal catalysts for olefin insertion polymerization and copolymerization. Chem. Commun..

[6] Ye Z., Xu L., Dong Z., Xiang P. (2013). Designing polyethylenes of complex chain architectures via Pd-diimine-catalyzed “living” ethylene polymerization. Chem. Commun..

[7] Guan Z., Cotts P.M., McCord E.F., McLain S.J. (1999). Chain walking: A new strategy to control polymer topology. Science.

[8] Chen C.L., Luo S., Jordan R.F. (2008). Multiple insertion of a silyl vinyl ether by (α-diimine)PdMe^+^ species. J. Am. Chem. Soc..

[9] Chen C.L., Luo S., Jordan R.F. (2010). Cationic polymerization and insertion chemistry in the reactions of vinyl ethers with (α-diimine)PdMe^+^ species. J. Am. Chem. Soc..

[10] Chen C.L., Jordan R.F. (2010). Palladium-catalyzed dimerization of vinyl ethers to acetals. J. Am. Chem. Soc..

[11] Vaidya T., Klimovica K., LaPointe A.M., Keresztes I., Lobkovsky E.B., Daugulis O., Coates G.W. (2014). Secondary alkene insertion and precision chain-walking: A new route to semicrystalline “polyethylene” from α-olefins by combining two rare catalytic events. J. Am. Chem. Soc..

[12] Takano S., Takeuchi D., Osakada K., Akamatsu N., Shishido A. (2014). Dipalladium catalyst for olefin polymerization: Introduction of acrylate units into the main chain of branched polyethylene. Angew. Chem. Int. Ed..

[13] Wang R.K., Sui X.L., Pang W.M., Chen C.L. (2015). Ethylene polymerization by xanthene bridged dinuclear α-diimine Ni(II) complexes. Chem. Cat. Chem..

[14] Guo L.H., Chen C.L. (2015). (α-Diimine) palladium catalyzed ethylene polymerization and copolymerization with polar comonomers. Sci. China Chem..

[15] Guo L.H., Dai S.Y., Sui X.L., Chen C.L. (2016). Palladium and nickel catalyzed chain walking olefin polymerization and copolymerization. ACS Catal..

[16] Xie T.Y., Mcauley K.B., Hsu J.C.C., Bacon D.W. (1994). Gas phase ethylene polymerization: Production processes, polymer properties, and reactor modeling. Ind. Eng. Chem. Res..

[17] Tempel D.J., Johnson L.K., Huff R.L., White P.S., Brookhart M. (2000). Mechanistic studies of Pd(II)-α-diimine-catalyzed olefin polymerizations. J. Am. Chem. Soc..

[18] Gates D.P., Svejda S.A., Oñate E., Killian C.M., Johnson L.K., White P.S., Brookhart M. (2000). Synthesis of branched polyethylene using (α-diimine)nickel(II) catalysts: Influence of temperature, ethylene pressure, and ligand structure on polymer properties. Macromolecules.

[19] Berkefeld A., Mecking S. (2009). Deactivation pathways of neutral Ni(II) polymerization catalysts. J. Am. Chem. Soc..

[20] Guo L., Gao H., Guan Q., Hu H., Deng J., Liu J., Liu F., Wu Q. (2012). Substituent effects of the backbone in α-diimine palladium catalysts on homo- and copolymerization of ethylene with methyl acrylate. Organometallics.

[21] Ionkin A.S., Marshall W.J. (2004). *ortho*-5-Methylfuran- and benzofuran-substituted η^3^-allyl(α-diimine)nickel(II) complexes: Syntheses, structural characterization, and the first polymerization results. Organometallics.

[22] Camacho D.H., Salo E.V., Ziller J.W., Guan Z. (2004). Cyclophane-based highly active late-transition-metal catalysts for ethylene polymerization. Angew. Chem. Int. Ed..

[23] Camacho D.H., Guan Z. (2005). Living polymerization of α-olefins at elevated temperatures catalyzed by a highly active and robust cyclophane-based Nickel catalyst. Macromolecules.

[24] Popeney C.S., Camacho D.H., Guan Z. (2007). Efficient incorporation of polar comonomers in copolymerizations with ethylene using a cyclophane-based Pd(II) α-diimine catalyst. J. Am. Chem. Soc..

[25] Popeney C.S., Guan Z. (2009). A mechanistic investigation on copolymerization of ethylene with polar monomers using a cyclophane-based Pd(II) α-diimine catalyst. J. Am. Chem. Soc..

[26] Popeney C.S., Rheingold A.L., Guan Z. (2009). Nickel(II) and Palladium(II) polymerization catalysts bearing a fluorinated cyclophane ligand: Stabilization of the reactive intermediate. Organometallics.

[27] Liu F., Hu H., Xu Y., Guo L., Zai S., Song K., Gao H., Zhang L., Zhu F., Wu Q. (2009). Thermostable α-diimine Nickel(II) catalyst for ethylene polymerization: Effects of the Substituted backbone structure on catalytic properties and branching structure of polyethylene. Macromolecules.

[28] Kong S., Guo C.Y., Yang W., Wang L., Sun W.H., Glaser R. (2013). 2,6-Dibenzhydryl-*N*-(2-phenyliminoacenaphthylenylidene)-4-chloro-aniline nickel dihalides: Synthesis, characterization and ethylene polymerization for polyethylenes with high molecular weights. J. Organomet. Chem..

[29] Kong S., Song K., Liang T., Guo C.Y., Sun W.H., Redshaw C. (2013). Methylene-bridged bimetallic nickel(II) α-diimino nickel(II) complexes: Synthesis and high efficiency in ethylene polymerization. Dalton Trans..

[30] Liu H., Sun W.H. (2011). 2,6-Dibenzhydryl-*N*-(2-phenyliminoacenaphthylenylidene)-4-methylbenzenamine nickel dibromides: Synthesis, characterization, and ethylene polymerization. Organometallics.

[31] Rhinehart J.L., Brown L.A., Long B.K. (2013). A robust Ni(II) α-diimine catalyst for high temperature ethylene polymerization. J. Am. Chem. Soc..

[32] Rhinehart J.L., Mitchell N.E., Long B.K. (2014). Enhancing α-diimine catalysts for high-temperature ethylene polymerization. ACS Catal..

[33] Dai S.Y., Sui X.L., Chen C.L. (2015). Highly robust Pd(II) α–diimine catalysts for slow-chain-walking polymerization of ethylene and copolymerization with methyl acrylate. Angew. Chem. Int. Ed..

[34] Popeney C.S., Guan Z. (2005). Ligand electronic effects on late transition metal polymerization catalysts. Organometallics.

[35] Popeney C.S., Guan Z. (2010). Effect of ligand electronics on the stability and chain transfer rates of substituted Pd(II) α-diimine catalysts. Macromolecules.

[36] Popeney C.S., Levins C.M., Guan Z. (2011). Systematic investigation of ligand substitution effects in cyclophane-based nickel(II) and palladium(II) olefin polymerization catalysts. Organometallics.

[37] Moody L.S., Mackenzie P.B., Killian C.M., Lavoie G.G., Ponasik J.A., Barrett A.G., Smith T.W., Pearson J.C. (2002). Catalysts Containing *N*-pyrrolyl Substituted Nitrogen Donors.

[38] Meinhard D., Wegner M., Kipiani G., Hearley A., Reuter P., Fischer S., Marti O., Rieger B. (2007). New Nickel(II) diimine complexes and the control of polyethylene microstructure by catalyst design. J. Am. Chem. Soc..

