# Supplementary material for: Investigations of the Ligand Electronic Effects on α-Diimine Nickel(II) Catalyzed Ethylene Polymerization
Source: Polymers (Basel). 2016 Jan 29;8(2):37. doi: 10.3390/polym8020037 (PMC6432586; doi:10.3390/polym8020037)
Supplement: Supplementary file 1 [file polymers-08-00037-s001.pdf]

Supplementary Materials: Investigations of the Ligand Electronic Effects on α-Diimine Nickel (II) Catalyzed Ethylene Polymerization

Lihua Guo, Shengyu Dai and Changle Chen

Content

^{1}H NMR, ^{13}C NMR, ^{19}F NMR of the Nickel (II) Complexes. S2–S6

MALDI-TOF-MS and HRMS of Complexes **1a–1c**. S6–S7

GPC Curves of Polyethylene Generated by Complexes **2a–2d** at 100 °C. S8–S11

DSC Curves of Polyethylene Generated by Complexes **2a–2d** at 100 °C. S12–S13

^{1}H NMR of Polyethylene Generated by Complexes **2a–2d** at 100 °C. S14–S15

X-ray Crystallography of Complex **1a**. S16–S19

^{1}H NMR, ^{13}C NMR, ^{19}F NMR of the Nickel (II) Complexes.

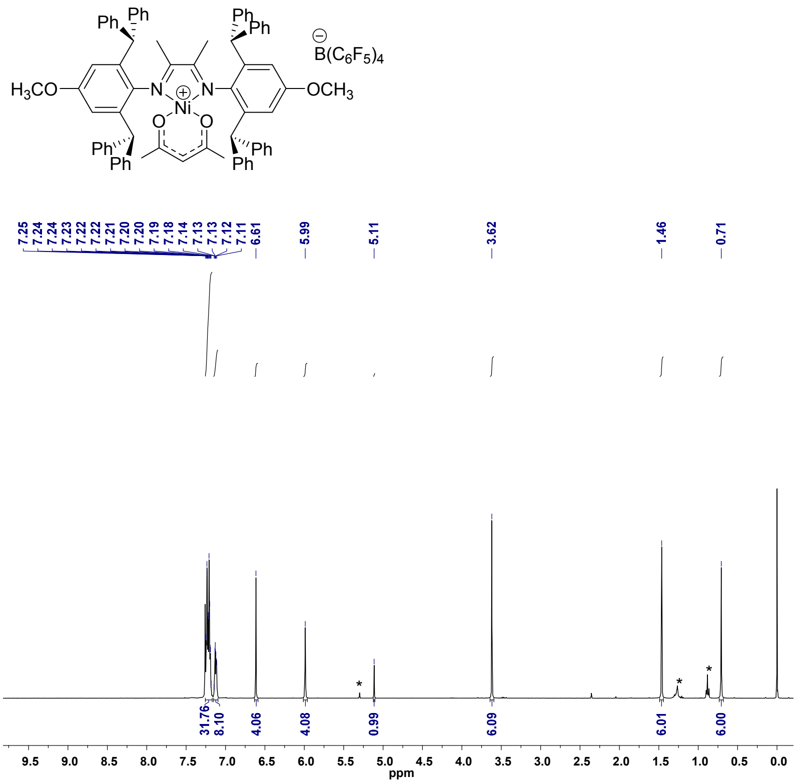

Figure S1. ^{1}H NMR spectrum (400 MHz) of **2a** in $CDCl_3$. * CH_2Cl_2, Hexane.

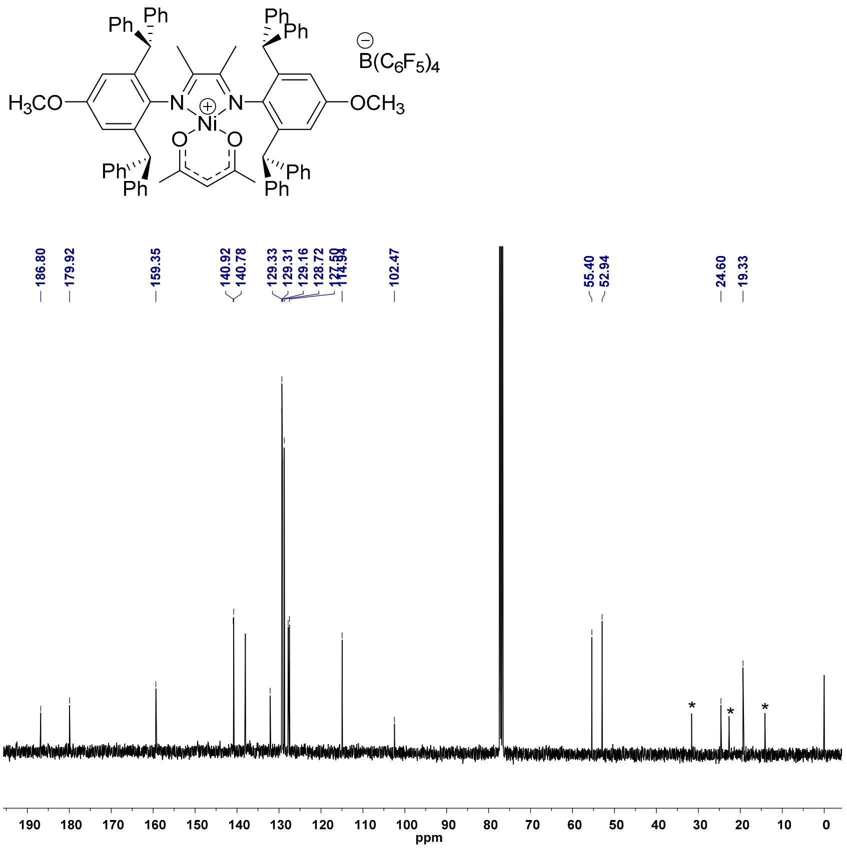

Figure S2. ^{13}C NMR spectrum (100 MHz) of **2a** in $CDCl_3$. * CH_2Cl_2, Hexane.

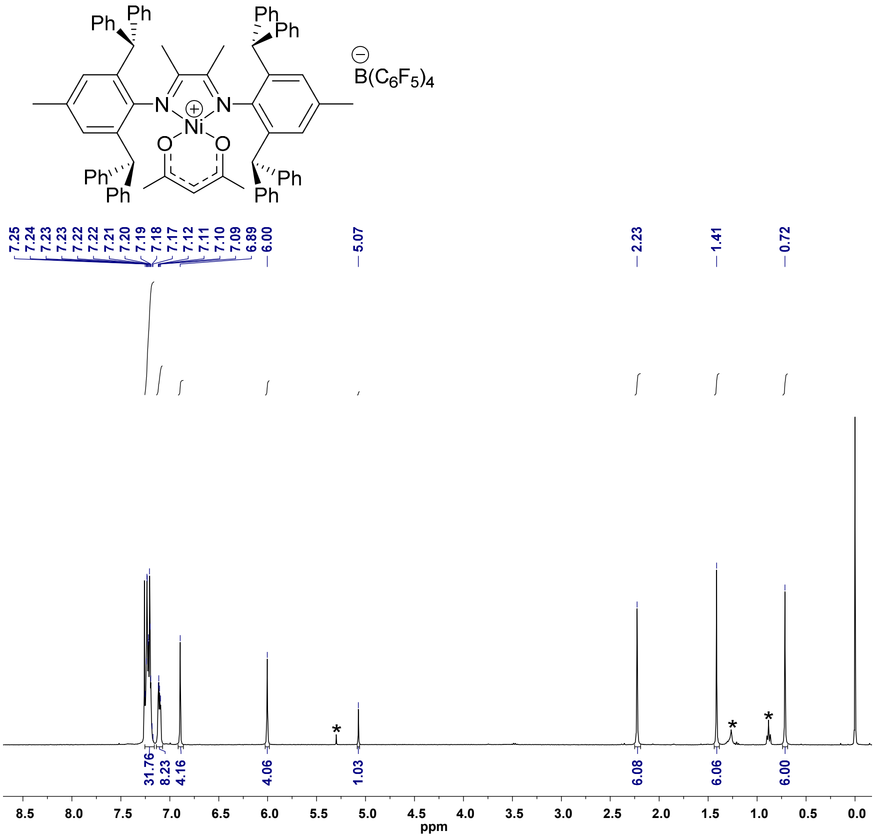

Figure S3. ^{1}H NMR spectrum (400 MHz) of **2b** in $CDCl_3$.* CH_2Cl_2, Hexane.

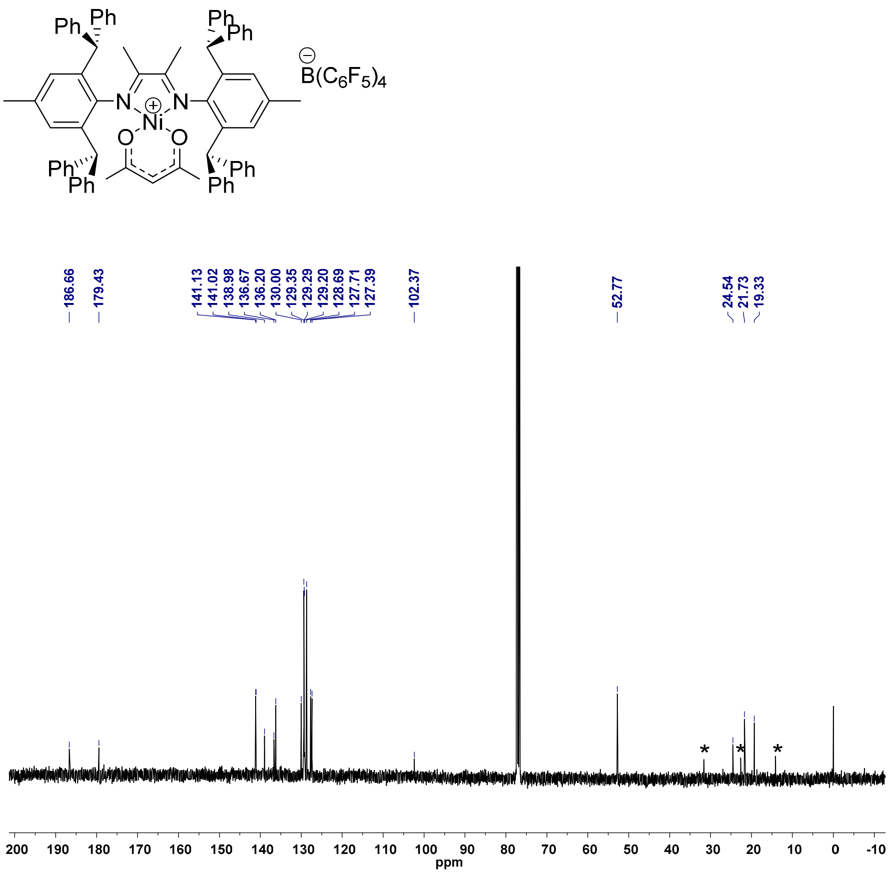

Figure S4. ^{13}C NMR spectrum (100 MHz) of **2b** in $CDCl_3$. * CH_2Cl_2, Hexane.

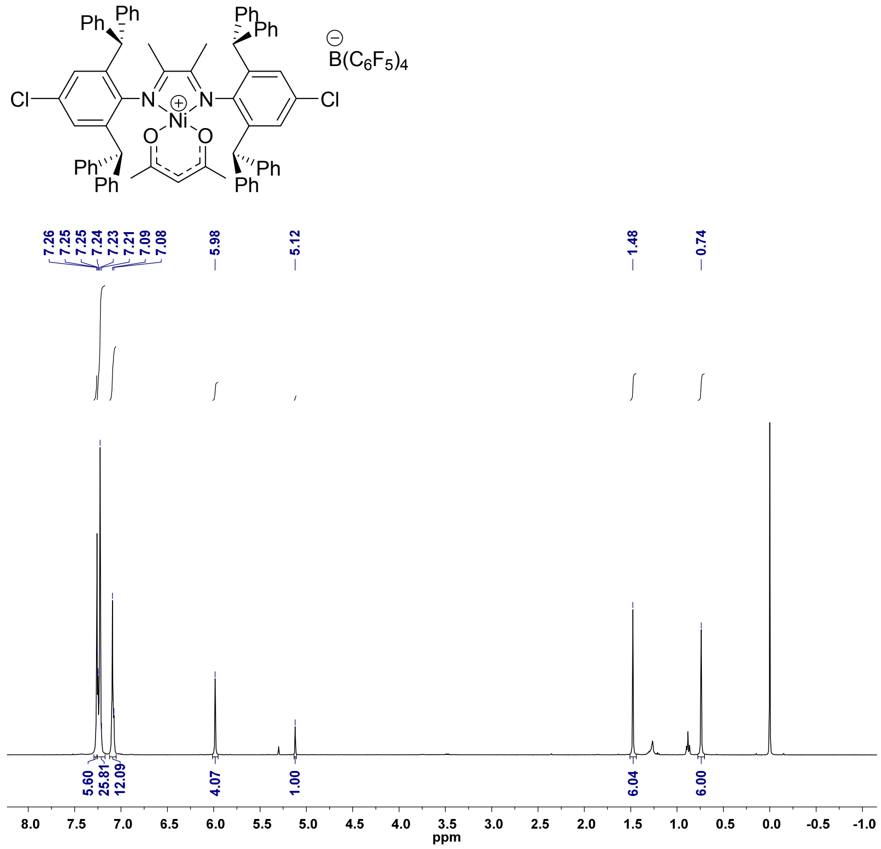

Figure S5. ^{1}H NMR spectrum (400 MHz) of **2c** in $CDCl_3$.

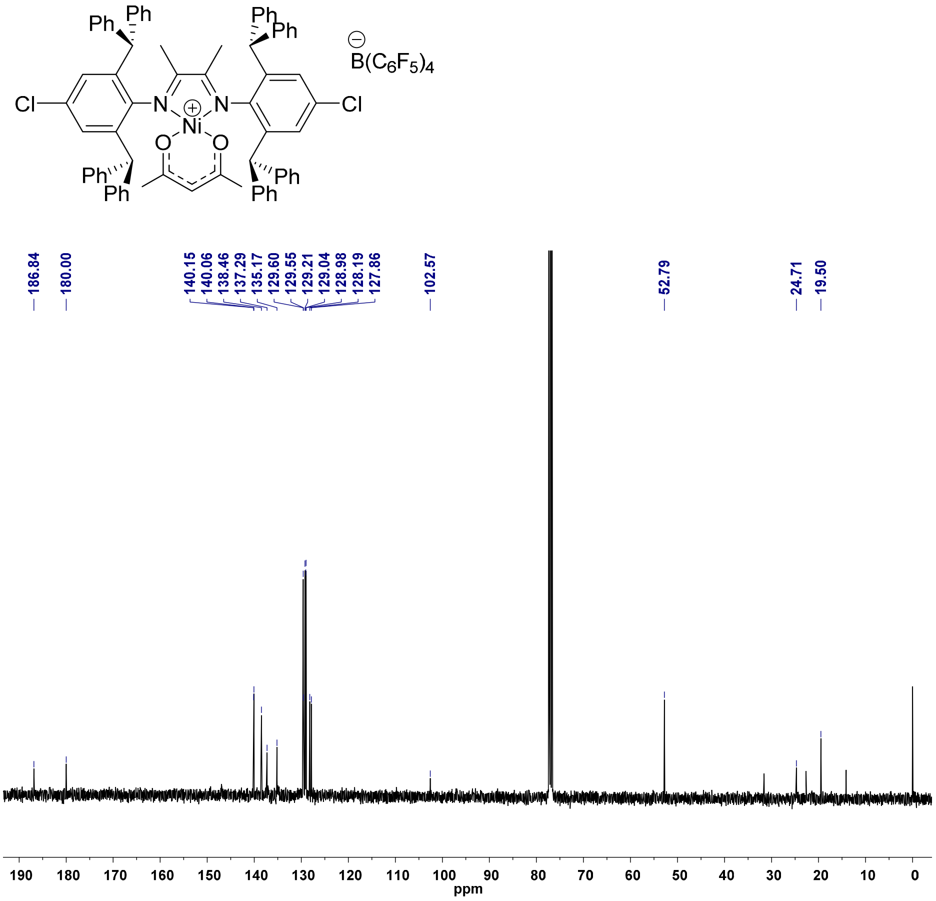

Figure S6. ^{13}C NMR spectrum (100 MHz) of **2c** in $CDCl_3$.

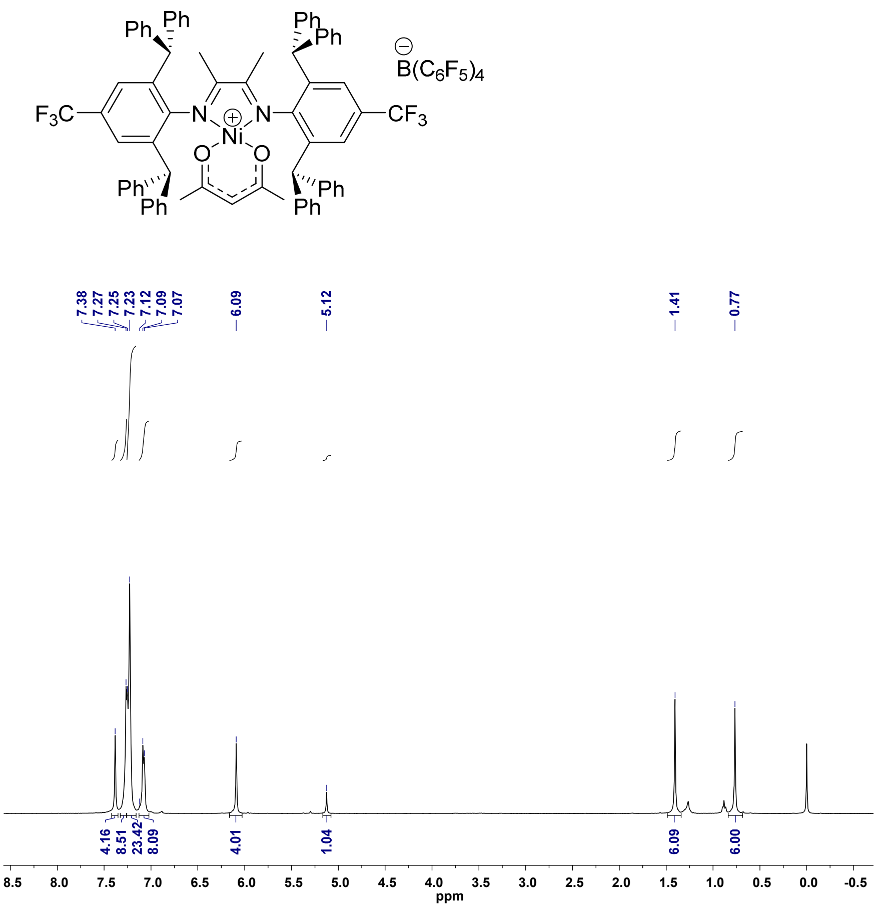

Figure S7. ^{1}H NMR spectrum (400 MHz) of **2d** in $CDCl_3$.

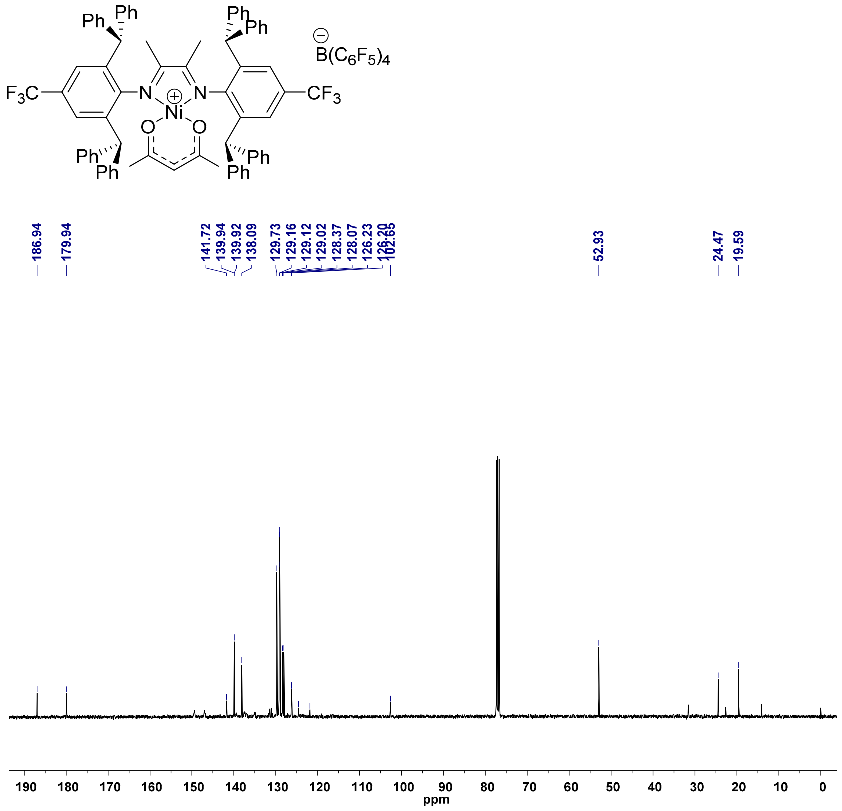

Figure S8. ^{13}C NMR spectrum (100 MHz) of 2d in $CDCl_3$.

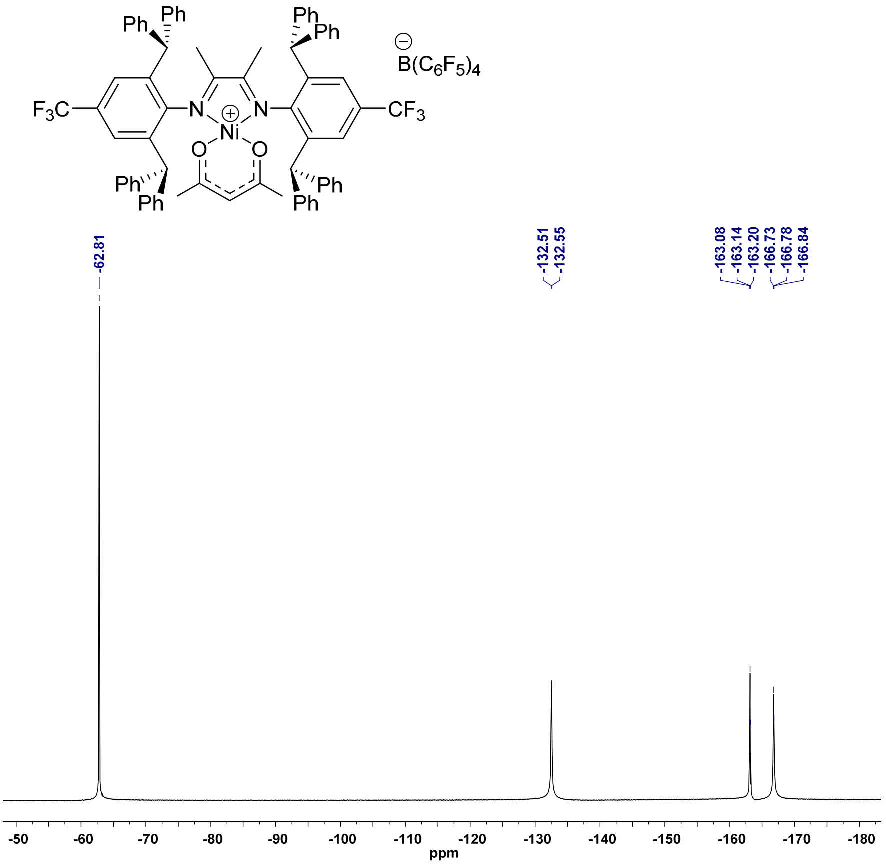

Figure S9. ^{19}F NMR spectrum (282 MHz) of **2d** in $CDCl_3$.

MALDI-TOF-MS and HRMS of Complexes 1a–1c.

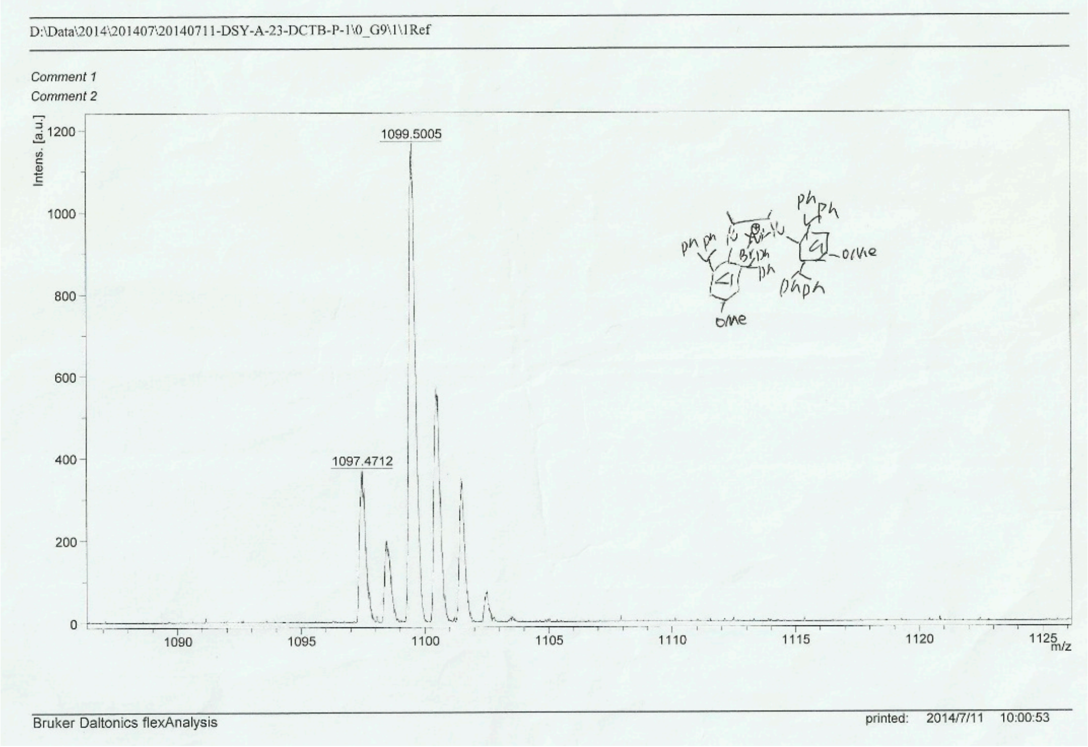

Figure S10. MALDI-TOF-MS of complex **1a**.

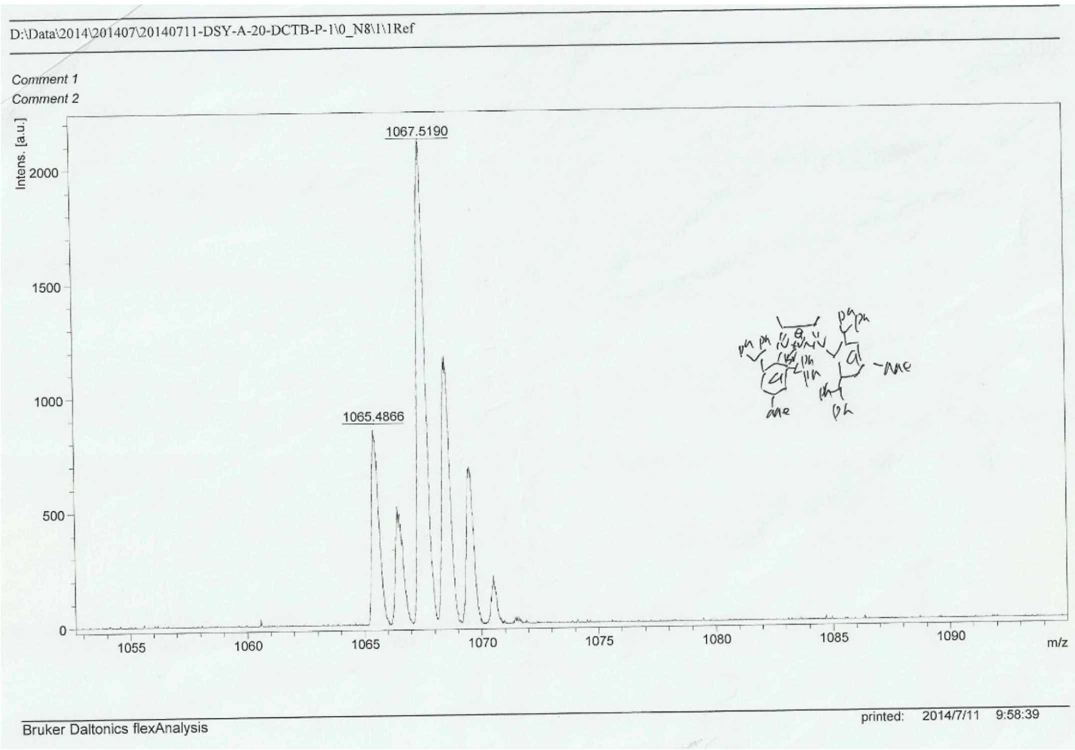

Figure S11. MALDI-TOF-MS of complex **1b**.

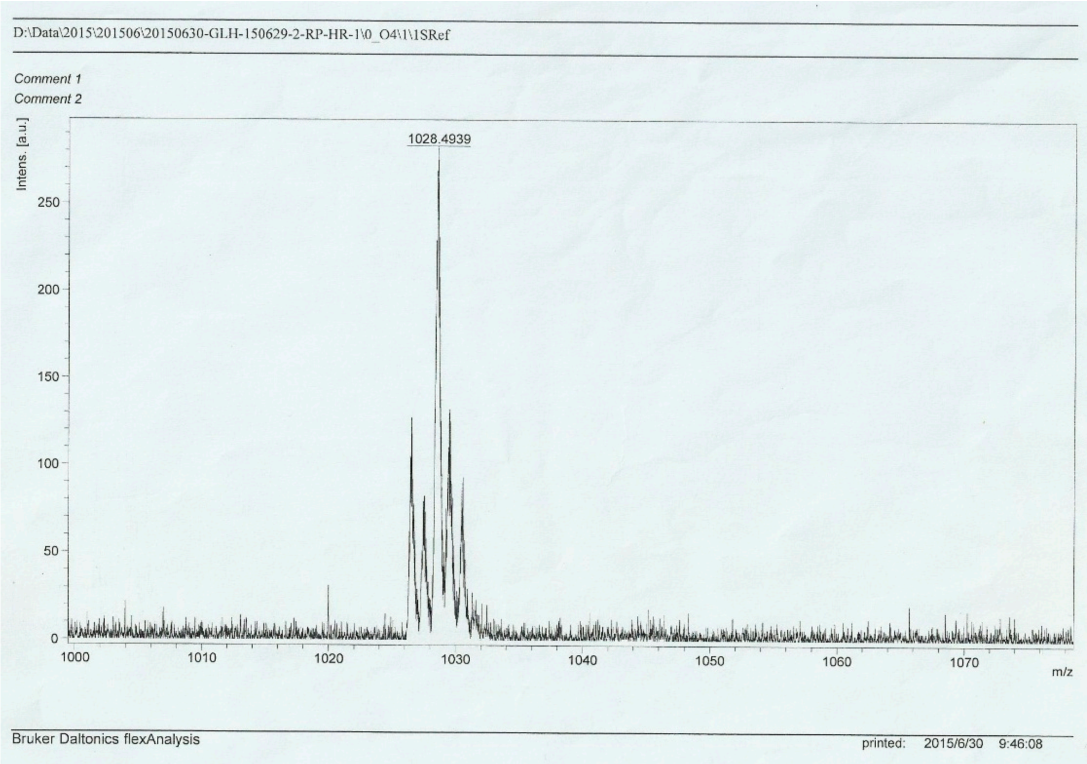

Figure S12. HRMS of complex **1c**.

GPC Curves of Polyethylene Generated by Complexes 2a–2d at 100 °C.

Cirrus GPC Sample Injection Report

Generated by: PLGPC 2015年11月22日 10:06

Workbook: D:\Cirrus Workbooks\20150208\20150208.plw

Sample Details

Sample Name: glh-74-1

Acquired: 2015-11-22 9:57:09 By Analyst: PLGPC Batch Name: Imported

Concentration: 0.10 mg/mlInjection Volume: 200.0 ul K of Sample: 14.1000 Alpha of Sample: 0.7000

Analysis Using Method: RIonly

Calibration Used: 2015-4-11 11:09:17

Calibration Type: Narrow Standard Curve Fit Used: 1 K: 14.1000 Alpha: 0.7000

Calibration Curve: y = 12.910720 - 0.578798x^1

High Limit MW RT: 11.32 mins Low Limit MW RT: 16.96 mins

Flow Marker RT: 0.00 mins FRCF: 1.0000 FRM Name:

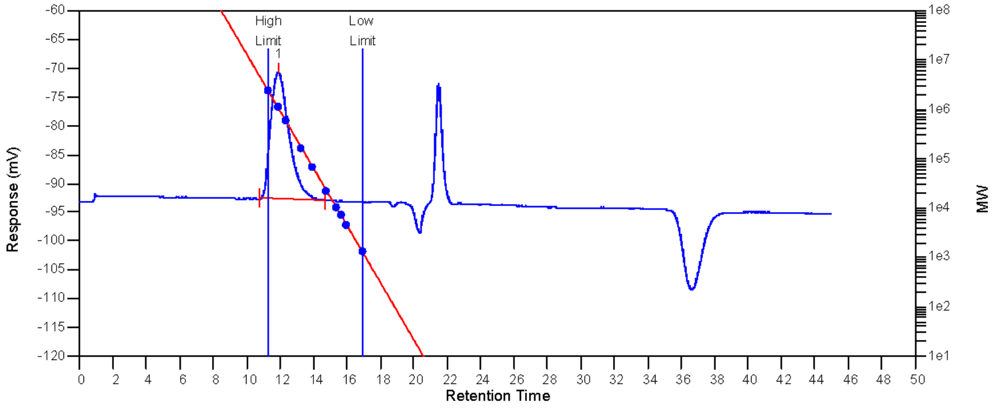

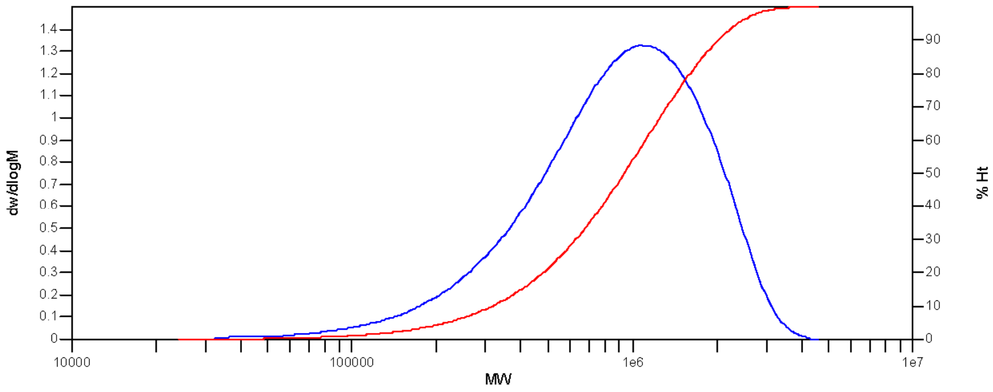

MW Averages

Peak No	Mp	Mn	Mw	Mz	Mz+1	Mv	PD
1	1066213	606312	1063029	1482004	1841235	1000735	1.75327

Processed Peaks

Peak No	Name	Start RT (mins)	Max RT (mins)	End RT (mins)	Pk Height (mV)	% Height	Area (mV.secs)	% Area
1		10.79	11.89	14.73	21.9941	0	1717.92	100

Cirrus GPC Version 3.2 Page 1 2015-11-22 10:06

Figure S13. GPC curves of Polyethylene generated by complex **2a**.

Cirrus GPC Sample Injection Report

Generated by: PLGPC 2015年11月22日 10:12

Workbook: D:\Cirrus Workbooks\20150208\20150208.plw

Sample Details

Sample Name: glh-74-2

Acquired: 2015-11-22 9:57:09 By Analyst: PLGPC Batch Name: Imported

Concentration: 0.10 mg/mlInjection Volume: 200.0 ul K of Sample: 14.1000 Alpha of Sample: 0.7000

Analysis Using Method: RIonly

Calibration Used: 2015-4-11 11:09:17

Calibration Type: Narrow Standard Curve Fit Used: 1 K: 14.1000 Alpha: 0.7000

Calibration Curve: y = 12.910720 - 0.578798x^1

High Limit MW RT: 11.32 mins Low Limit MW RT: 16.96 mins

Flow Marker RT: 0.00 mins FRCF: 1.0000 FRM Name:

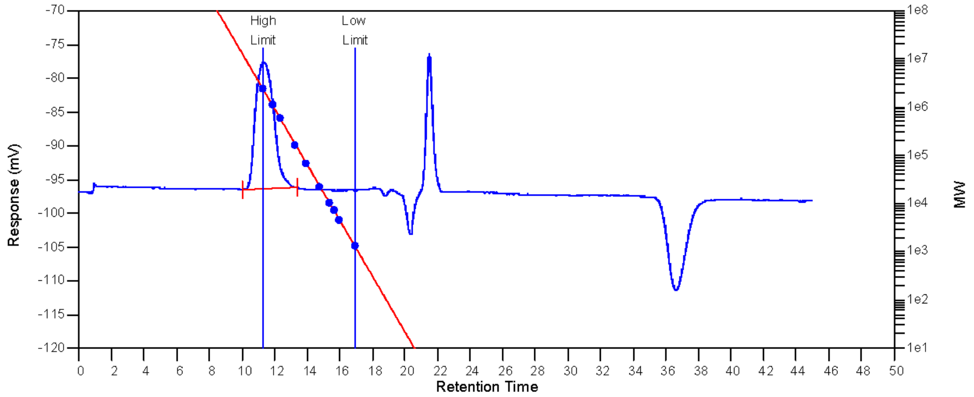

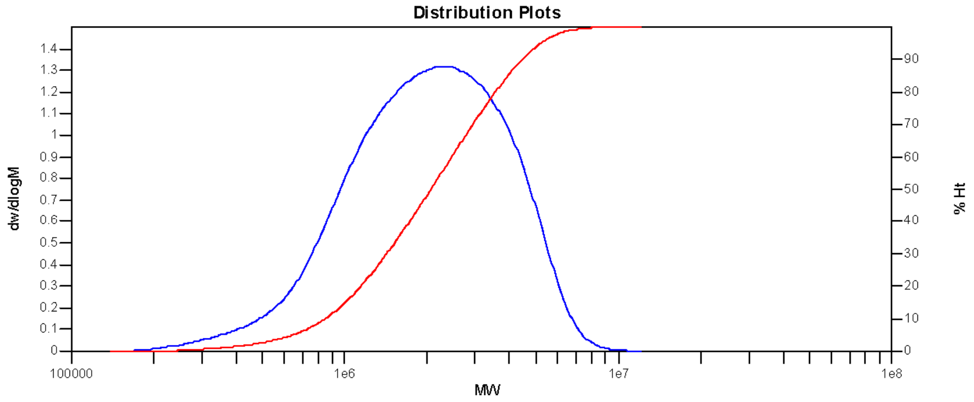

MW Averages

Peak No	Mp	Mn	Mw	Mz	Mz+1	Mv	PD
1	2398537	1569053	2378456	3236961	4026733	2255600	1.51585

Processed Peaks

Peak No	Name	Start RT (mins)	Max RT (mins)	End RT (mins)	Pk Height (mV)	% Height	Area (mV.secs)	% Area
1		10.07	11.28	13.42	18.8516	0	1479.81	100

Cirrus GPC Version 3.2 Page 1 2015-11-22 10:12

Figure S14. GPC curves of Polyethylene generated by complex **2b**.

Cirrus GPC Sample Injection Report

Generated by: PLGPC 2015年11月22日 10:14

Workbook: D:\Cirrus Workbooks\20150208\20150208.plw

Sample Details

Sample Name: glh-74-3

Acquired: 2015-11-22 9:57:09 By Analyst: PLGPC Batch Name: Imported

Concentration: 0.10 mg/mlInjection Volume: 200.0 ul K of Sample: 14.1000 Alpha of Sample: 0.7000

Analysis Using Method: RIonly

Calibration Used: 2015-4-11 11:09:17

Calibration Type: Narrow Standard Curve Fit Used: 1 K: 14.1000 Alpha: 0.7000

Calibration Curve: y = 12.910720 - 0.578798x^1

High Limit MW RT: 11.32 mins Low Limit MW RT: 16.96 mins

Flow Marker RT: 0.00 mins FRCF: 1.0000 FRM Name:

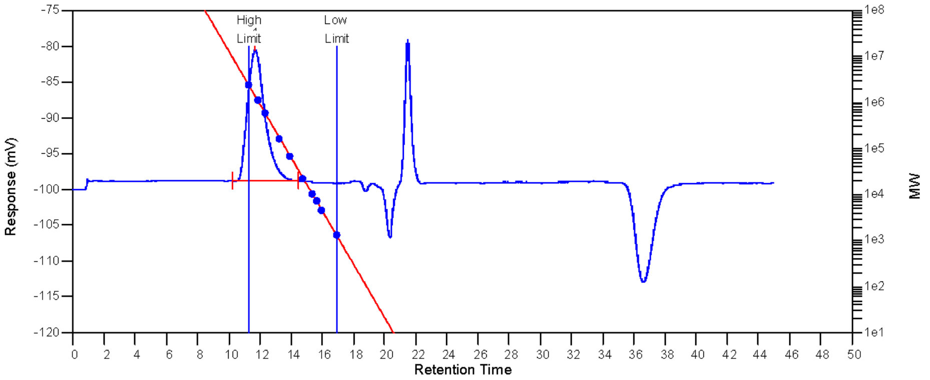

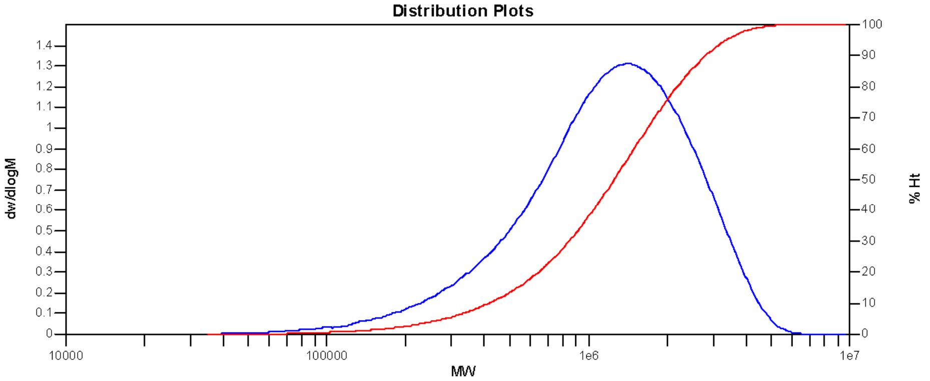

MW Averages

Peak No	Mp	Mn	Mw	Mz	Mz+1	Mv	PD
1	1423149	809625	1452410	2073881	2632245	1362299	1.79393

Processed Peaks

Peak No	Name	Start RT (mins)	Max RT (mins)	End RT (mins)	Pk Height (mV)	% Height	Area (mV.secs)	% Area
1		10.24	11.68	14.46	18.3122	0	1446.65	100

Cirrus GPC Version 3.2 Page 1 2015-11-22 10:14

Figure S15. GPC curves of Polyethylene generated by complex **2c**.

Cirrus GPC Sample Injection Report

Generated by: PLGPC 2015年11月22日 10:16

Workbook: D:\Cirrus Workbooks\20150208\20150208.plw

Sample Details

Sample Name: glh-74-4

Acquired: 2015-11-22 9:57:09 By Analyst: PLGPC Batch Name: Imported

Concentration: 0.10 mg/mlInjection Volume: 200.0 ul K of Sample: 14.1000 Alpha of Sample: 0.7000

Analysis Using Method: RIonly

Calibration Used: 2015-4-11 11:09:17

Calibration Type: Narrow Standard Curve Fit Used: 1 K: 14.1000 Alpha: 0.7000

Calibration Curve: y = 12.910720 - 0.578798x^1

High Limit MW RT: 11.32 mins Low Limit MW RT: 16.96 mins

Flow Marker RT: 0.00 mins FRCF: 1.0000 FRM Name:

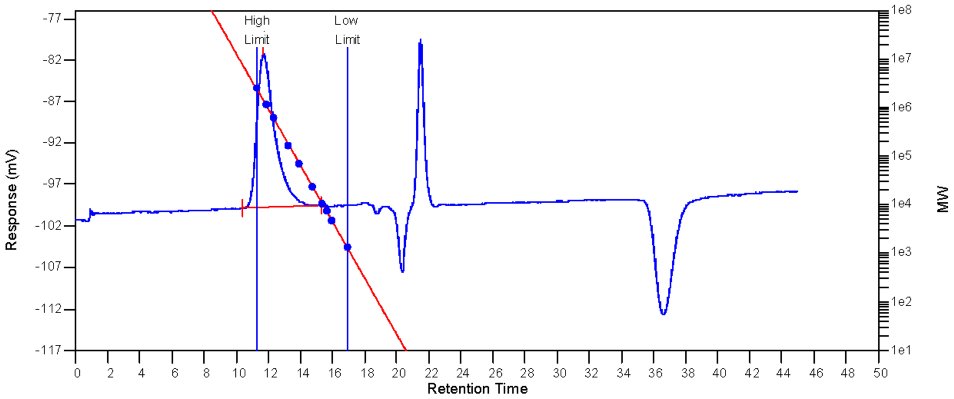

Distribution Plots

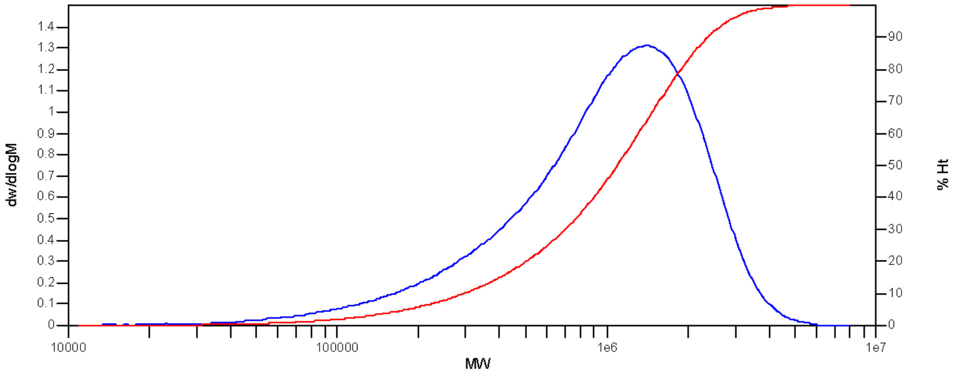

MW Averages

Peak No	Mp	Mn	Mw	Mz	Mz+1	Mv	PD
1	1423149	575356	1234014	1788824	2266109	1150277	2.14478

Processed Peaks

Peak No	Name	Start RT (mins)	Max RT (mins)	End RT (mins)	Pk Height (mV)	% Height	Area (mV.secs)	% Area
1		10.38	11.68	15.33	18.5892	0	1469.34	100

Cirrus GPC Version 3.2 Page 1 2015-11-22 10:16

Figure S16. GPC curves of Polyethylene generated by complex **2d**.

DSC Curves of Polyethylene Generated by Complexes 2a–2d at 100 °C.

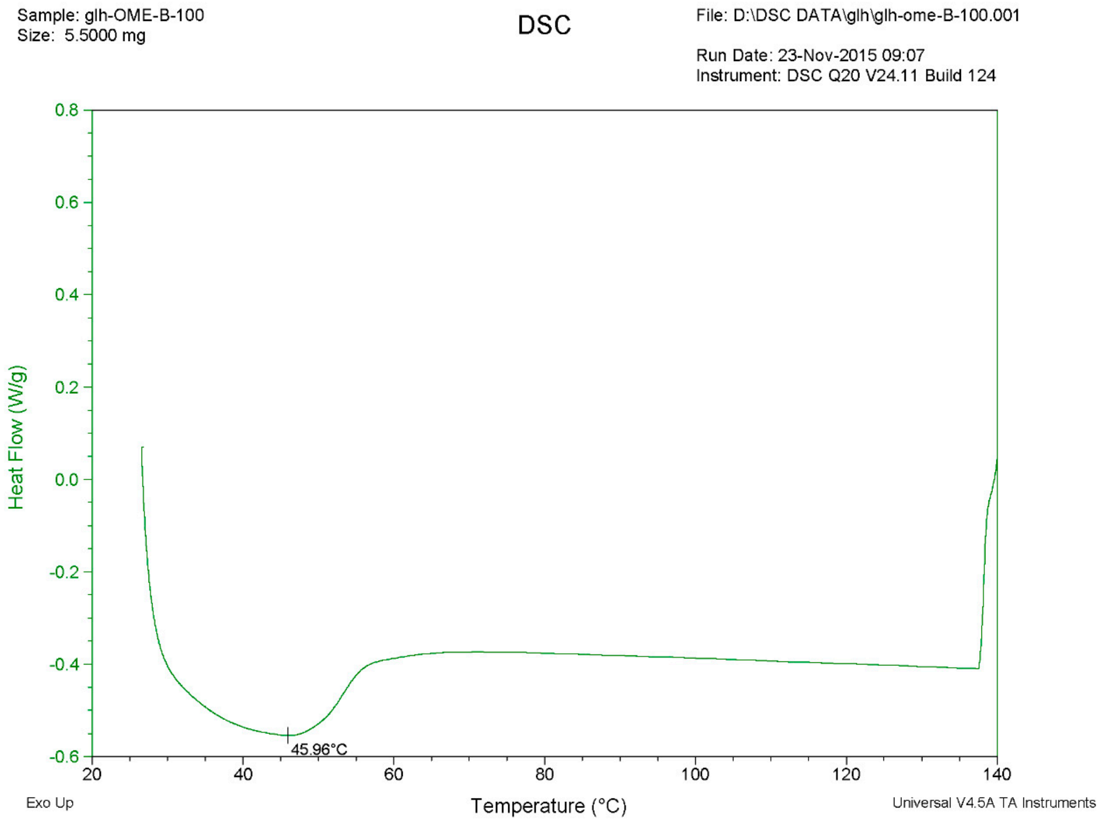

Figure S17. DSC of Polyethylene generated by complex **2a** (Table 2, Entry 10).

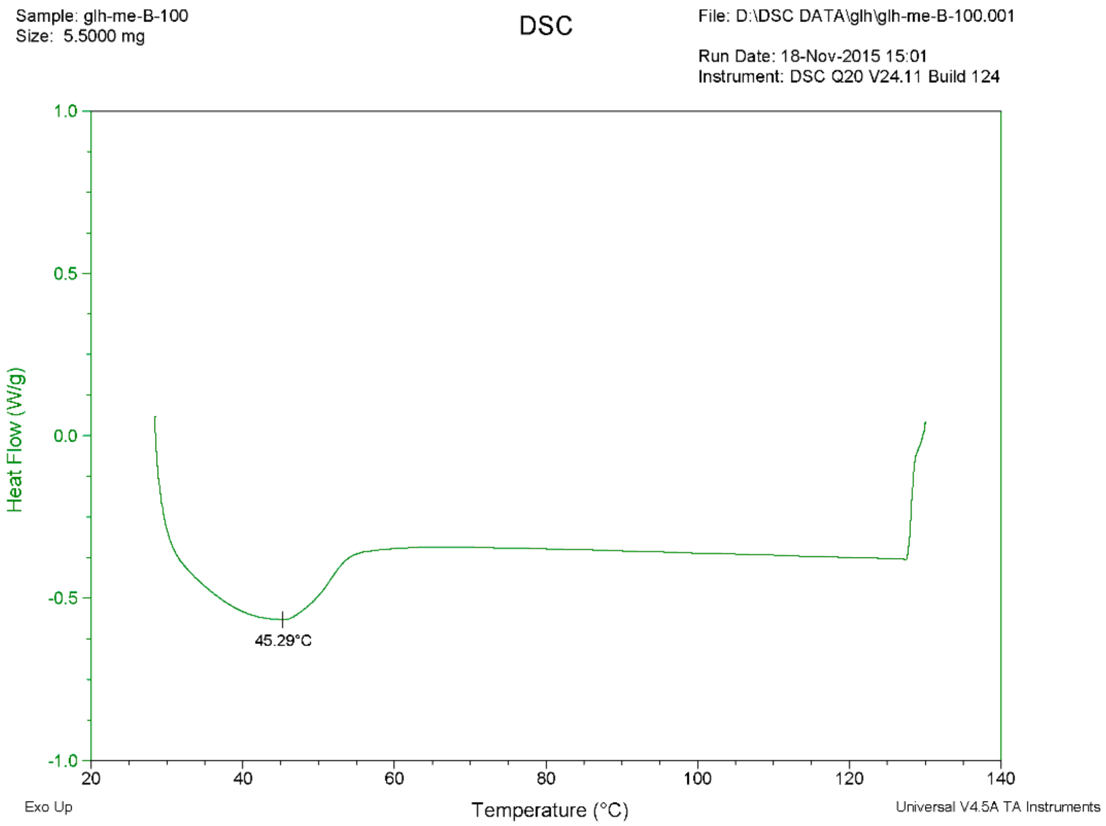

Figure S18. DSC of Polyethylene generated by complex **2b** (Table 2, Entry 9).

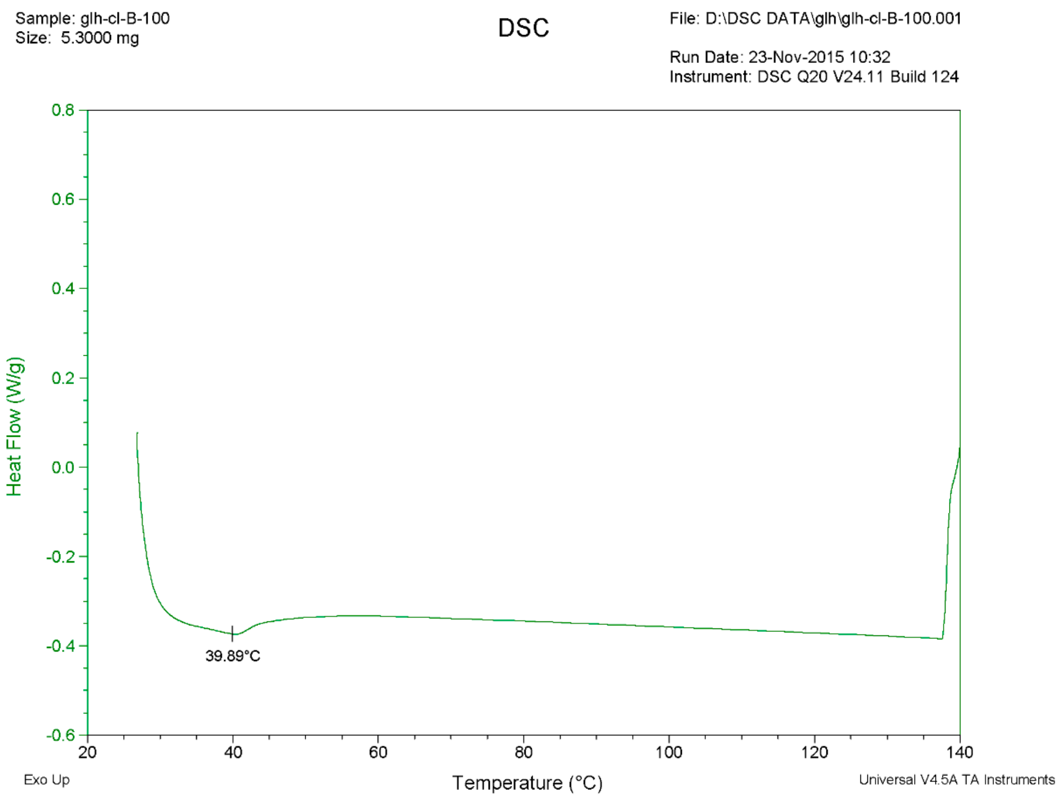

Figure S19. DSC of Polyethylene generated by complex **2c** (Table 2, Entry 11).

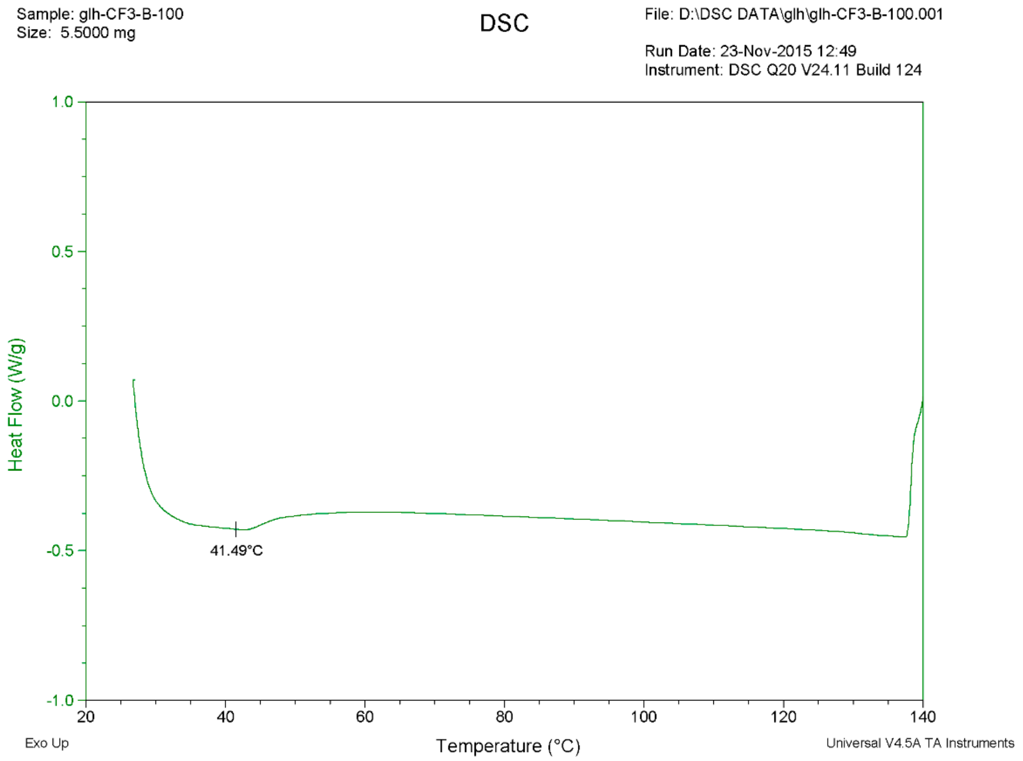

Figure S20. DSC of Polyethylene generated by complex **2d** (Table 3, Entry 12).

^{1}H NMR of Polyethylene Generated by Complexes 2a–2d at 100 °C.

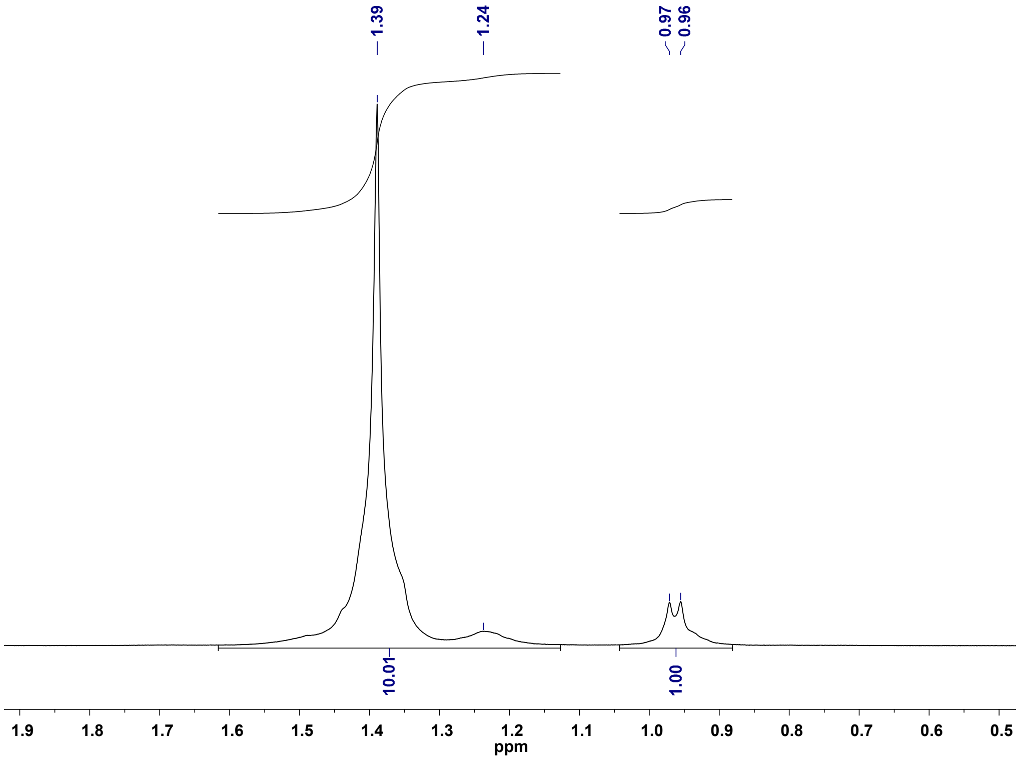

Figure S21. ^{1}H NMR of Polyethylene generated by complex **2a** (Table 2, Entry 10).

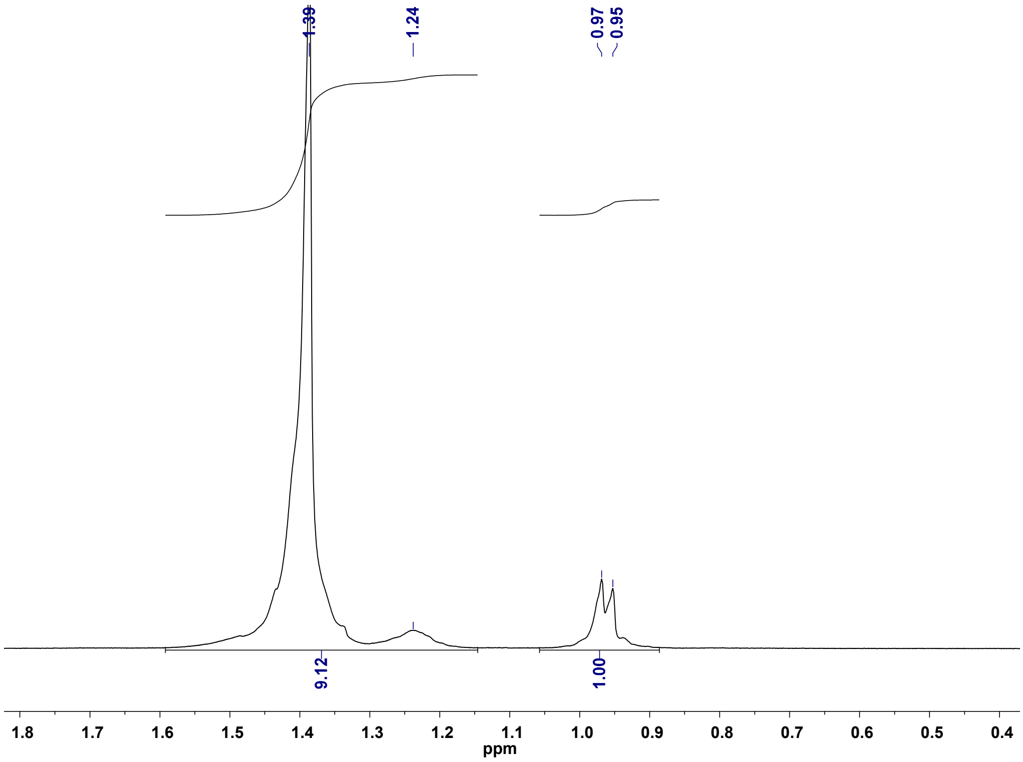

Figure S22. ^{1}H NMR of Polyethylene generated by complex **2b** (Table 2, Entry 9).

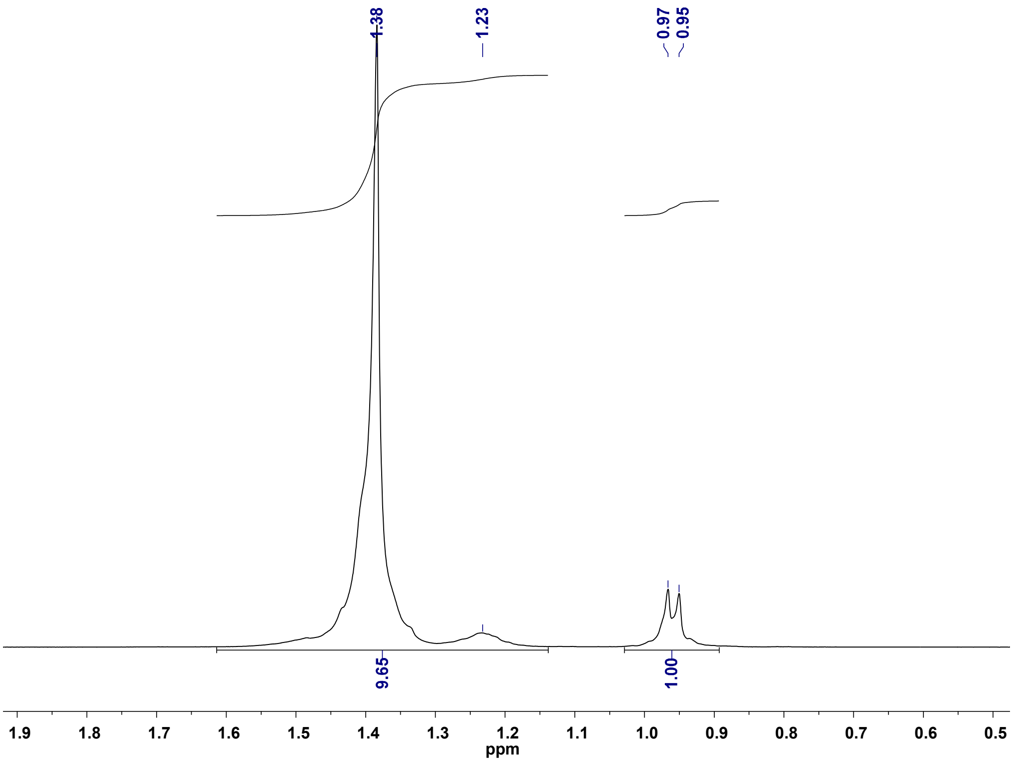

Figure S23. ^{1}H NMR of Polyethylene generated by complex **2c** (Table 2, Entry 11).

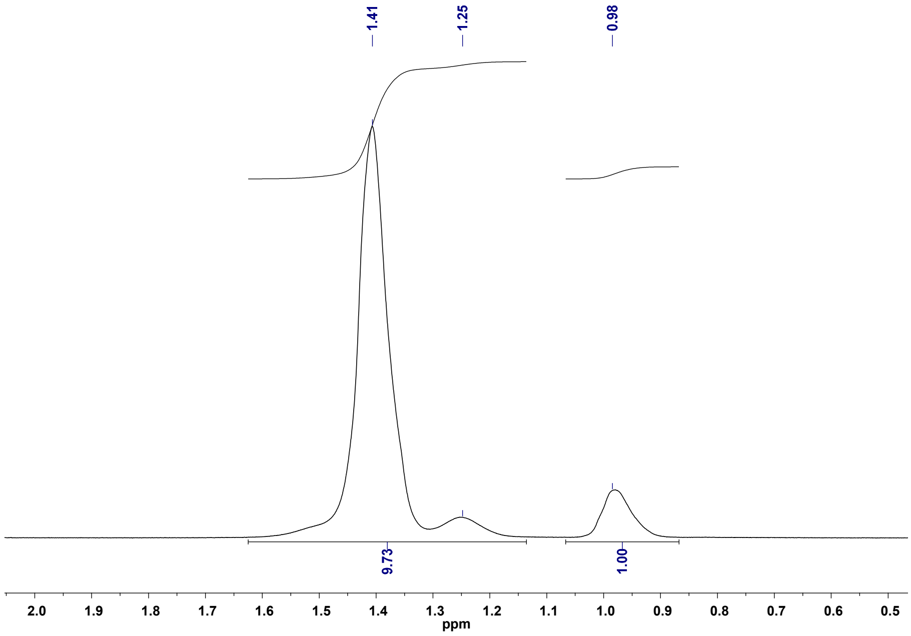

Figure S23. ^{1}H NMR of Polyethylene generated by complex **2c** (Table 2, Entry 12).

X-ray Crystallography of Complex 1a

CCDC number of **1a** is 1442213. These data can be obtained free of charge from The Cambridge Crystallographic Data Centre via www.ccdc.cam.ac.uk/data_request/cif.

Table S1. Crystal data and structure refinement for **1a**.

Identification code	1a
Empirical formula	$C_{71}H_{64}Br_2Cl_2N_2NiO_3$
Formula weight	1282.67
Temperature/K	295 (2)
Crystal system	monoclinic
Space group	$P2_1/c$
a/Å	18.625 (2)
b/Å	18.902 (3)
c/Å	17.707 (2)
α/°	90
β/°	100.248 (2)
γ/°	90
Volume/Å^3	6134.4 (14)
Z	4
ρ_{calc}g/cm^3	1.389
μ/mm^{-1}	1.755
F (000)	2640.0
Crystal size/mm^3	0.46 × 0.40 × 0.32
Radiation	MoKα (λ = 0.71073)
2θ range for data collection/°	2.222 to 55.906
Index ranges	$-24 \leq h \leq 23$, $-24 \leq k \leq 24$, $-23 \leq l \leq 22$
Reflections collected	48,418
Independent reflections	13510 [R_{int} = 0.1360, R_{sigma} = 0.1828]
Data/restraints/parameters	13,510/0/738
Goodness-of-fit on F^2	0.960
Final R indexes [$I \geq 2\sigma(I)$]	R_1 = 0.0743, wR_2 = 0.1783
Final R indexes [all data]	R_1 = 0.2159, wR_2 = 0.2270
Largest diff. peak/hole/e Å^{-3}	0.94/−0.76

Table S2. Bond Lengths for **1a**.

Atom	Atom	Length/Å	Atom	Atom	Length/Å
Ni1	N2	1.986 (5)	C34	C35	1.350 (13)
Ni1	N1	1.988 (5)	C35	C36	1.300 (13)
Ni1	Br1	2.3058 (12)	C36	C37	1.360 (13)
Ni1	Br2	2.3163 (11)	C38	C39	1.388 (8)
C1	C2	1.483 (9)	C38	C57	1.388 (8)
C2	N1	1.272 (7)	C38	N1	1.463 (8)
C2	C3	1.501 (8)	C39	C53	1.392 (9)
C3	N2	1.291 (7)	C39	C40	1.517 (8)
C3	C4	1.481 (8)	C40	C47	1.510 (9)
C5	C6	1.409 (8)	C40	C41	1.532 (8)
C5	C24	1.413 (8)	C41	C46	1.359 (9)
C5	N2	1.453 (7)	C41	C42	1.388 (10)
C6	C20	1.383 (8)	C42	C43	1.361 (10)
C6	C7	1.499 (8)	C43	C44	1.378 (12)
C7	C8	1.522 (8)	C44	C45	1.358 (11)
C7	C14	1.527 (8)	C45	C46	1.381 (10)
C8	C13	1.379 (9)	C47	C52	1.374 (10)
C8	C9	1.386 (9)	C47	C48	1.381 (10)
C9	C10	1.356 (10)	C48	C49	1.404 (11)
C10	C11	1.353 (11)	C49	C50	1.384 (12)
C11	C12	1.362 (11)	C50	C51	1.338 (13)
C12	C13	1.391 (10)	C51	C52	1.334 (12)
C14	C19	1.360 (9)	C53	C54	1.374 (9)
C14	C15	1.373 (9)	C54	C56	1.367 (9)
C15	C16	1.372 (10)	C54	O1	1.375 (8)
C16	C17	1.344 (11)	C55	O1	1.412 (9)
C17	C18	1.357 (10)	C56	C57	1.375 (9)
C18	C19	1.383 (9)	C57	C58	1.516 (8)
C20	C21	1.392 (8)	C58	C59	1.522 (10)
C21	O2	1.369 (7)	C58	C65	1.530 (9)
C21	C23	1.370 (8)	C59	C64	1.369 (10)
C22	O2	1.407 (7)	C59	C60	1.395 (10)
C23	C24	1.380 (8)	C60	C61	1.367 (13)
C24	C25	1.532 (8)	C61	C62	1.387 (17)
C25	C26	1.531 (10)	C62	C63	1.335 (16)
C25	C32	1.532 (10)	C63	C64	1.340 (14)
C26	C27	1.351 (11)	C65	C66	1.373 (10)
C26	C31	1.369 (10)	C65	C70	1.387 (9)
C27	C28	1.369 (12)	C66	C67	1.371 (11)
C28	C29	1.345 (14)	C67	C68	1.364 (12)
C29	C30	1.334 (13)	C68	C69	1.330 (12)
C30	C31	1.402 (12)	C69	C70	1.360 (11)
C32	C33	1.330 (11)	C71	Cl1	1.598 (14)
C32	C37	1.372 (10)	C71	Cl2	1.754 (15)
C33	C34	1.417 (12)			

Table S3. Bond Angles for **1a**.

Atom	Atom	Atom	Angle/°	Atom	Atom	Atom	Angle/°
N2	Ni1	N1	81.1 (2)	C36	C37	C32	121.1 (9)
N2	Ni1	Br1	113.25 (14)	C39	C38	C57	120.8 (6)
N1	Ni1	Br1	104.59 (15)	C39	C38	N1	119.9 (5)
N2	Ni1	Br2	109.91 (14)	C57	C38	N1	118.9 (5)
N1	Ni1	Br2	116.79 (14)	C38	C39	C53	119.5 (6)
Br1	Ni1	Br2	123.45 (5)	C38	C39	C40	121.9 (6)
N1	C2	C1	126.9 (6)	C53	C39	C40	118.4 (6)
N1	C2	C3	114.4 (6)	C47	C40	C39	111.5 (6)
C1	C2	C3	118.7 (6)	C47	C40	C41	111.5 (5)
N2	C3	C4	125.8 (6)	C39	C40	C41	112.5 (5)
N2	C3	C2	115.3 (6)	C46	C41	C42	117.8 (7)
C4	C3	C2	118.9 (6)	C46	C41	C40	122.9 (7)
C6	C5	C24	121.4 (5)	C42	C41	C40	119.4 (6)
C6	C5	N2	117.7 (5)	C43	C42	C41	121.7 (7)
C24	C5	N2	120.8 (5)	C42	C43	C44	119.1 (9)
C20	C6	C5	118.3 (5)	C45	C44	C43	120.2 (8)
C20	C6	C7	121.0 (5)	C44	C45	C46	119.7 (8)
C5	C6	C7	120.6 (5)	C41	C46	C45	121.3 (8)
C6	C7	C8	111.0 (5)	C52	C47	C48	116.6 (8)
C6	C7	C14	114.8 (5)	C52	C47	C40	123.6 (7)
C8	C7	C14	111.1 (5)	C48	C47	C40	119.8 (7)
C13	C8	C9	117.4 (7)	C47	C48	C49	121.2 (8)
C13	C8	C7	122.6 (6)	C50	C49	C48	117.7 (9)
C9	C8	C7	120.0 (6)	C51	C50	C49	120.7 (10)
C10	C9	C8	121.6 (7)	C52	C51	C50	120.6 (10)
C11	C10	C9	121.0 (8)	C51	C52	C47	123.0 (9)
C10	C11	C12	119.2 (8)	C54	C53	C39	118.9 (6)
C11	C12	C13	120.7 (8)	C56	C54	C53	121.5 (6)
C8	C13	C12	120.2 (7)	C56	C54	O1	114.7 (6)
C19	C14	C15	117.1 (6)	C53	C54	O1	123.8 (7)
C19	C14	C7	119.8 (6)	C54	C56	C57	120.5 (6)
C15	C14	C7	122.8 (6)	C56	C57	C38	118.8 (6)
C16	C15	C14	120.9 (7)	C56	C57	C58	118.8 (6)
C17	C16	C15	121.4 (8)	C38	C57	C58	122.3 (6)
C16	C17	C18	118.7 (8)	C57	C58	C59	112.0 (6)
C17	C18	C19	120.2 (8)	C57	C58	C65	113.3 (6)
C14	C19	C18	121.6 (7)	C59	C58	C65	111.5 (6)
C6	C20	C21	120.6 (6)	C64	C59	C60	118.8 (8)
O2	C21	C23	114.6 (6)	C64	C59	C58	122.3 (8)
O2	C21	C20	124.9 (6)	C60	C59	C58	118.9 (8)
C23	C21	C20	120.4 (6)	C61	C60	C59	119.6 (10)
C21	C23	C24	121.6 (6)	C60	C61	C62	119.3 (12)
C23	C24	C5	117.8 (6)	C63	C62	C61	120.1 (14)
C23	C24	C25	118.0 (5)	C62	C63	C64	121.4 (14)
C5	C24	C25	124.1 (6)	C63	C64	C59	120.8 (10)
C26	C25	C24	110.4 (6)	C66	C65	C70	115.7 (7)
C26	C25	C32	112.2 (6)	C66	C65	C58	120.7 (7)
C24	C25	C32	113.1 (6)	C70	C65	C58	123.5 (7)
C27	C26	C31	118.5 (8)	C67	C66	C65	121.7 (8)
C27	C26	C25	120.3 (7)	C68	C67	C66	120.2 (10)

Table S3. *Cont.*

Atom	Atom	Atom	Angle/°	Atom	Atom	Atom	Angle/°
C31	C26	C25	121.2 (8)	C69	C68	C67	119.3 (10)
C26	C27	C28	121.4 (10)	C68	C69	C70	120.9 (9)
C29	C28	C27	120.7 (12)	C69	C70	C65	122.0 (8)
C30	C29	C28	118.9 (12)	Cl1	C71	Cl2	114.0 (8)
C29	C30	C31	121.7 (10)	C2	N1	C38	122.6 (6)
C26	C31	C30	118.7 (9)	C2	N1	Ni1	115.0 (4)
C33	C32	C37	118.3 (8)	C38	N1	Ni1	122.5 (4)
C33	C32	C25	122.8 (7)	C3	N2	C5	121.8 (5)
C37	C32	C25	118.5 (8)	C3	N2	Ni1	113.9 (4)
C32	C33	C34	119.4 (9)	C5	N2	Ni1	124.3 (4)
C35	C34	C33	120.1 (10)	C54	O1	C55	116.6 (6)
C36	C35	C34	119.7 (11)	C21	O2	C22	118.8 (5)
C35	C36	C37	121.4 (10)				

© 2016 by the authors; licensee MDPI, Basel, Switzerland. This article is an open access article distributed under the terms and conditions of the Creative Commons by Attribution (CC-BY) license (http://creativecommons.org/licenses/by/4.0/).
